# Social Health Inequalities and eHealth: A Literature Review With Qualitative Synthesis of Theoretical and Empirical Studies

**DOI:** 10.2196/jmir.6731

**Published:** 2017-04-27

**Authors:** Karine Latulippe, Christine Hamel, Dominique Giroux

**Affiliations:** ^1^ Department of Studies of Teaching and Learning Laval University Québec, QC Canada; ^2^ Faculté de médecine Département de réadaptation Laval University Québec, QC Canada; ^3^ Centre d'Excellence du Vieillissement de Québec, Chu de Québec Québec, QC Canada

**Keywords:** Internet, social media, telemedecine, healthcare disparities

## Abstract

**Background:**

eHealth is developing rapidly and brings with it a promise to reduce social health inequalities (SHIs). Yet, it appears that it also has the potential to increase them.

**Objectives:**

The general objective of this review was to set out how to ensure that eHealth contributes to reducing SHIs rather than exacerbating them. This review has three objectives: (1) identifying characteristics of people at risk of experiencing social inequality in health; (2) determining the possibilities of developing eHealth tools that avoid increasing SHI; and (3) modeling the process of using an eHealth tool by people vulnerable to SHI.

**Methods:**

Following the EPPI approach (Evidence for Policy and Practice of Information of the Institute of Education at the University of London), two databases were searched for the terms SHIs and eHealth and their derivatives in titles and abstracts. Qualitative, quantitative, and mixed articles were included and evaluated. The software NVivo (QSR International) was employed to extract the data and allow for a metasynthesis of the data.

**Results:**

Of the 73 articles retained, 10 were theoretical, 7 were from reviews, and 56 were based on empirical studies. Of the latter, 40 used a quantitative approach, 8 used a qualitative approach, 4 used mixed methods approach, and only 4 were based on participatory research-action approach. The digital divide in eHealth is a serious barrier and contributes greatly to SHI. Ethnicity and low income are the most commonly used characteristics to identify people at risk of SHI. The most promising actions for reducing SHI via eHealth are to aim for universal access to the tool of eHealth, become aware of users’ literacy level, create eHealth tools that respect the cultural attributes of future users, and encourage the participation of people at risk of SHI.

**Conclusions:**

eHealth has the potential to widen the gulf between those at risk of SHI and the rest of the population. The widespread expansion of eHealth technologies calls for rigorous consideration of interventions, which are not likely to exacerbate SHI.

## Introduction

### Background

A number of studies have demonstrated that eHealth is effective in preventing and treating illness for the entire population [1-6]. eHealth is the way to improve health care locally, regionally, and worldwide by using information and communication technology [7]. At a political level, the American Recovery Reinvestment Act authorized the government to spend US $38 billion over 10 years to develop eHealth in order to increase accessibility to care [8]. Australia’s National eHealth Strategy predicts that eHealth will transform the manner in which consumers interact with the health care system and will lead to a reduction in costs and demands on the system [9]. According to Health Canada [10], eHealth is an essential element in the renewal of health care and its application to the Canadian system, thanks to improvements in accessibility, and the quality and efficiency of the system; this is beneficial to Canadians. However, eHealth also has the potential to increase social health inequalities (SHIs) [6,11-13]. SHIs, such as the difference in the prevalence of illness and of illness repercussions, the mortality rate, and the burden of illness and other health conditions for specific population groups are caused by unjust and modifiable social factors [14]. This term includes inequalities and inequities in the environment, access, utilization and the quality of services, health status, and the results of interventions [15]. Indeed, eHealth is effective to the extent that individuals are in a position to use it well. Yet, this is not the case for everyone; in fact, this creates a gap between users and nonusers in terms of the improvement of health services.

The reduction of SHI is a key challenge for health systems worldwide, including in Canada, and eHealth is an economical and political means to that end. Yet, since it also has the potential to increase them, it is essential to focus on developing eHealth tools that, in fact, contribute to the reduction of SHI and not their exacerbation. This leads to the following question: how do we ensure that eHealth contributes to reducing social inequalities rather than exacerbating them? In responding to the research question, we have three objectives: (1) identifying characteristics of people at risk of experiencing social inequality in health; (2) determining the possibilities of developing eHealth tools that avoid increasing SHI; and (3) modeling the process of using an eHealth tool by people vulnerable to SHI. To answer this question, a review of the literature is required.

### eHealth Tool and SHIs

Previous literature reviews on the relationship between SHIs and eHealth are summarized here to both incorporate already existing knowledge on the subject and to demonstrate the relevance and contribution of this review. Seven reviews on the relationship between SHI and eHealth were identified over the last decade. First, Gibbons et al [16] reviewed some design principles based on solid data to improve the facility with which people at risk of SHI handle eHealth tools. They identified 5 principles to consider when developing an inclusive eHealth tool:

1. Use a design based on experimentation with the tool allowing us to identify the nature of possible errors and the strategies to employ.
2. Create a tool for people with limited resources in order to ensure that all users are readily able to use it.
3. Whenever possible, avoid authentication procedures with the tool (if this aspect is indispensable, considering the personal data that utilization of the tool requires, ensure that technical assistance is provided to users).
4. Minimize the potential of having harmful information inadvertently available.
5. Evaluate the tool with representative users.

For their part, the objective of Dorstyn et al [17] was to synthesize quantitative evidence related to the efficacy of adult telecounseling for a racial minority. They demonstrated the efficacy of this type of eHealth tool in comparison to monitoring alone, but this has yet to be proven in comparison to face-to-face encounters.

Next, Montague and Perchonok [18], in their review of the literature, examined how technology is used by historically disadvantaged populations to reduce health inequalities. Thus, they addressed four research questions: (1) What types of technologies were used to improve health results of historically disadvantaged populations? They discovered that videos, the Internet (including access via mobile phones), computers, and radios were the most studied technologies. (2) On what health problems is technology focused? The five most studied problems are cancer, diabetes, human immunodeficiency virus (HIV), nutrition, physical activity, and sexually transmitted infections. (3) For which historically disadvantaged groups have eHealth interventions been designed? 19 groups were identified in the literature, including Americans of African origin, Hispanics, indigenous people, and Americans of Asian origin. (4) How were the impacts of the use of such technologies evaluated? Self-evaluation measures are the most common, followed by physiological changes.

For their part, Huxley et al [19] attempted to understand the effects of interventions linked to digital communication in specific contexts (marginalized groups vs the general population). They revealed a number of barriers to the use of communication in general for marginalized groups including, notably, difficulties of access, and stigmatizing reactions from both health professionals and other patients. Nonetheless, digital communication has the potential to reduce these barriers by providing anonymity and offering advantages for those needing an interpreter. This form of communication is liable to function well when there is a preexisting relationship with the practitioner.

For their part, Chou and colleagues [20] explored the evidence concerning the use of Web 2.0 and social media and their impact on the promotion of health. From this, they concluded that the lack of empirical research meant that further investigation was required, especially concerning the design of tools accessible to vulnerable populations.

McInnes et al [21] studied access to and utilization of information technologies among the homeless. They found that use varied from 24% to 84%, depending on the technology (cellular, computer, or access to a public computer), and suggest that this technology could contribute to improving the health of this population.

Finally, Piette et al [22] conducted a scoping review to identify data on the effects of eHealth on health outcomes and costs. They conclude that “Although large programs for eHealth implementation and research are being conducted in many low- and middle-income countries, more information on the impacts of eHealth on outcomes and costs in these settings is still needed.”

Thus, although these reviews of the literature make a major contribution to the body of knowledge on the relationship between SHI and eHealth, they only partially address the question and research objectives. This review of the literature is intended to complement the reviews cited previously.

## Methods

### Approach

The EPPI (Evidence for Policy and Practice of Information of the Institute of Education at the University of London) approach was used in this procedure [23]. This approach suggests an iterative process with an explanation and a justification of the choices made. The EPPI approach offers an armory of tools and strategies for conducting research reviews on “how” to use eHealth tools to reduce SHI. The EPPI approach was chosen for its openness to integrating different types of studies and their variety of methodologies. It aims at the understanding of a phenomenon, to which every study, regardless of design, has the potential to contribute [24].

### Criteria of Inclusion and Exclusion

In order to respond adequately to the research questions, criteria of inclusion and exclusion were established. The criteria of inclusion were (1) articles published within the last decade (2006-2016); (2) in a peer-reviewed academic journal; (3) in English or French; (4) with an abstract available for screening by title and abstract; and (5) related to the research subject. For this last criterion, it was established that the article must concern eHealth and SHI; eHealth and the populations at risk of SHI (related to poverty, ethnicity, gender, mental health, age, low levels of literacy, HIV, low levels of numeracy, sexual orientation, rural residence, or tobacco addiction); or eHealth in the general population, but demonstrate inequality through a differentiated sociodemographic analysis. The four first criteria of inclusion were applied through research filters available from the databases. In the context of this review, the eHealth tools examined are those concerning education of the entire population or of individuals and do not include technological tools related to the management of the health care system, the monitoring of the health of the entire population, education for professionals, and the exchange of information between organizations.

Articles were excluded if the study focused on health or educational professionals, if the eHealth tool was exclusively a method to collect data for research, or if the article was not available.

### Research Strategy

There are countless knowledge transfer platforms related to eHealth and SHI. Nevertheless, to make this review as replicable as possible, it was decided that the references needed to be tracked by database. From April to July 2016, articles were identified from two databases related to the research subject, Medline (PubMed) and Cumulative Index to Nursing and Allied Health Literature (CINAHL). For each database, terms corresponding to key concepts as well as those associated with the thesaurus of each database were used and searched for in titles and abstracts. The terms corresponding to key concepts were identified from leading articles on eHealth and SHI, with the help of a specialist in documentation from the University of Laval.

The first chain of terms related to eHealth included: eHealth, Web-based, Internet, interactive health communication*, health communication*, computer communication network*, computer-assisted therapy, computer assisted, software, communication* media, telecommunication*, multimedia, medical information technolog*, computing, consumer* health information technolog*, World Wide Web, computer-assisted instruction*, interactive technolog* application*, hypermedia*, video game*, Virtual realit*, online learning, social media*, new media*, participatory media*, user-generated content, Facebook, MySpace, Twitter, YouTube, Second Life, wiki*, blog*, Web 2.0, online social network, social networking, health application*+thesaurus: (PubMed) Internet, social media; (CINHAL) and information science with the Boolean operator “OR” between each term.

The second chain of terms stemming from SHIs included underprivileged, health inequalit*, inequalit* in health, poverty, inequalit*, social inequalit*, socioeconomic inequalit*, health for all, health-related exclusion, health disparit*, health equit*, equit*, in health, vulnerable group*, inequalit*, disparit* in health+thesaurus: (PubMed) Health Care Disparities+socioeconomic factors+poverty; (CINAHL) Health Care Disparities+health status disparities+poverty, also with the Boolean operator “OR” between each term. Then, the two chains of concepts were interconnected with the Boolean operator “AND.”

The articles identified were exported to software for bibliographical references (Zotero) to facilitate the classification, importation, and exportation of documents, as well as the removal of duplicates. References were then imported using Covidence [25] (a Cochrane technology platform) to select articles respecting the criteria of inclusion and exclusion, first by titles and abstracts, and then by the complete article. Covidence was specifically designed to support systematic reviews.

### Evaluation of the Quality of Articles

To evaluate the quality of quantitative studies, the *Quality Assessment Tool for Quantitative Studies* [26] was used. This tool was judged to be excellent in evaluating the quality of studies in public health [27]. The qualitative studies were themselves evaluated based on an adaptation of quality standards from qualitative studies of Letts et al [28], including Guba and Lincoln [29] and Howe and Eisenhart [30]. There are no generally accepted norms by which to assess the methodological quality of mixed methods [31]. Nonetheless, we chose to use the criteria of Schifferdecker and Reed [32] to produce more precise guidelines. The reviews were evaluated by assessment of multiple systematic reviews (AMSTAR) [33], a valid and reliable instrument for evaluating the methodological quality of systematic reviews [27,34].

In order to meet the objectives of this review of the literature and to ensure that we were not eliminating data relevant to the research, the quality of articles was not evaluated with the goal of excluding articles but rather to consider their limitations in the course of the analysis and synthesis of knowledge [24].

### Analysis

The analysis was completed in two stages. First, thematic analysis [35], with the use of Nvivo software (QSR International) allowed for the classification of themes related to the research goals emerging from the articles retained. Thematic analysis permits us to identify all the relevant themes for our research [35]. The data were extracted from all the articles selected and organized, with an inductive approach, by theme, according in conjunction with the objectives of the review. The final themes selected are (1) characteristics of those studied who are at risk of SHI; (2) potential obstacles to the use of eHealth tools; (3) interventions in eHealth that could potentially contribute to the diminution of SHI; (4) eHealth interventions that could potentially contribute to the increase of SHI; and (5) the types of technology. Next, a metasynthesis was performed to enhance understanding of the creation of SHI in eHealth [24]. Metasynthesis serves to comprehend a phenomenon [36]. The analysis, with the help of conceptualizing categories, forms the basis of this metasynthesis [35]. A conceptual map was created with MindMaple Lite (MindMaple Inc) according to the interpretation of the articles in order to model the process of using an eHealth tool by people at risk of experiencing SHI. This map was then designed to facilitate comprehension. Each stage of the process, as well as the resulting choices and justifications, were documented in a logbook and supervised by the director (CH) of the principal author (KL). A general outline of the studies done (descriptive mapping) will first be presented.

## Results

### Articles Selected

**Figure 1:**
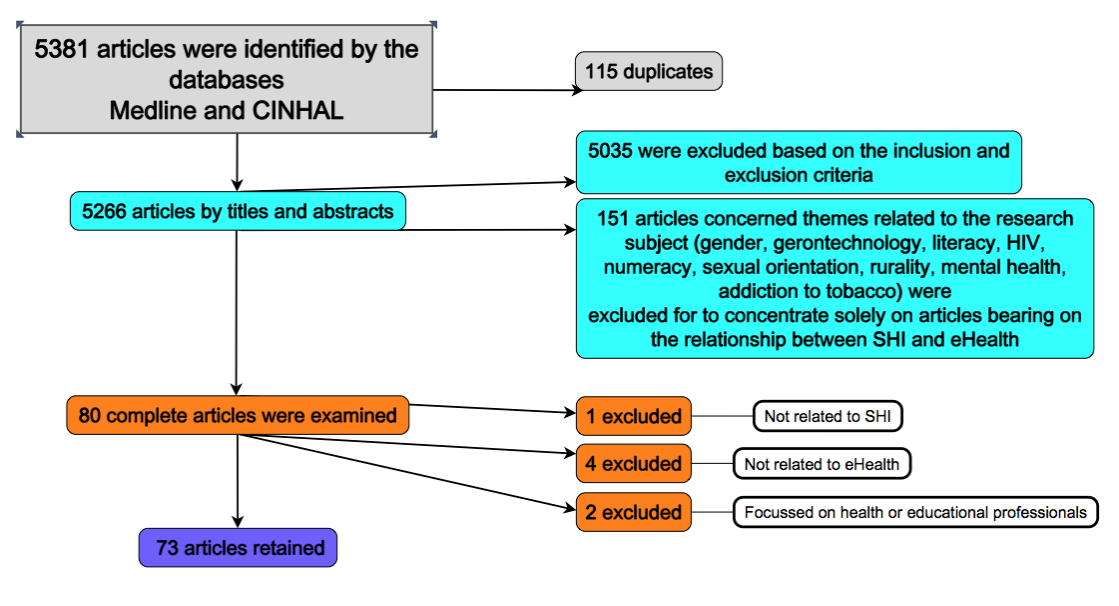
PRISMA flow diagram.

A total of 5381 articles were identified by the databases. Of this number, 115 duplicates were eliminated. Thus, the titles and abstracts of 5266 articles were first examined. It was found that 5035 were excluded based on the inclusion and exclusion criteria. Of the remaining number, 151 articles concerned themes related to the research subject (gender, gerontechnology, literacy, HIV, numeracy, sexual orientation, rurality, mental health, addiction to tobacco). Although these articles could contribute to the exploration of some principal themes, it was decided to concentrate solely on articles bearing on the relationship between SHI and eHealth. Thus, 80 complete articles were examined and, of these 7 were ultimately excluded, bringing the final number to 73 articles retained for this analysis (Figure 1).

### Description of Included Studies

Of the 73 articles retained, 10 were theoretical, 7 were reviews of the literature previously referenced, and 56 were empirical studies. Of the latter, 40 adopted a quantitative approach, 8 employed a qualitative approach, 4 used mixed methods, and 4 were based on participatory research action (see Multimedia Appendix 1). The majority of empirical studies were American (44), and the rest were British (4), Australian (4), Dutch (2), and German (1).

The quality of studies varied (Multimedia Appendix 2). However, the conceptual categories retained in the metasynthesis all recurred in the articles, which diminished the effect of the weaker studies.

A large proportion of the studies that focused on the utilization of technology in daily life among people at risk of SHI (15) attempt to comprehend how people at risk of SHI seek health information on the Internet (11) or how a specific eHealth tool is used by people at risk of SHI (10). Only three studies examined document the development of an eHealth tool for those in a situation of SHI. The other studies concern the evaluation of attrition in the use of an eHealth tool, education about the utilization of the technology, acceptance of a technology by this clientele, differences in the type of communication, accessibility, and confidentiality among people at risk of SHI. Another study focused on the development of a measure of SHI in eHealth. Finally, certain studies (3) evaluate the frequency of use and the rate of attrition of a technology for the population as a whole through a differentiated analysis of sociodemographic data (see Multimedia Appendix 1).

### Digital Divide and Social Health Inequalities

Unequal access to the Internet, the primary digital divide, has an effect on the utilization of eHealth [37]. The term “digital divide” first sends us back to the separation between those who have access to technologies such as computers, mobile phones, or the Internet and those who do not have access, often people with low income [6,11-13,38-42].

Although the digital divide still exists, it has diminished every year [43,44] with the use of mobile phones and other mobile devices [45], the reduced cost of technology and the Internet, as well as the spread of places where the Internet is free [40]. However, even though access is a crucial element in the utilization of a technology, this is not sufficient [12]. Thus, certain researchers have determined that knowledge related to the utilization of the Internet also has an impact. This knowledge gap between users is called the secondary digital divide [12,40,44,46-48]. Indeed, it is possible to have the capacity to connect to the Internet, but to lack sufficient knowledge to use it adequately. This highlights the need to develop new users’ skills, along with interventions to increase access [11,12,44].

Today, some authors are identifying other barriers to the utilization of technologies, referred to as the tertiary digital divide. Much more widespread, this tertiary digital divide refers to the concept of significant (or universal) access encompassing equipment, Internet connections, the development of skills, technical assistance, and appropriate content, that is, that health information be comprehensible and useful for disadvantaged populations [37,39-41,47]. In particular, this includes geographical location, literacy, attitudes and behavior with respect to the search for information, confidence and concerns about private life and institutional policies, and content, including the lack of local information, language, incapacities, and the lack of cultural sensitivity [39].

It is important to mention that the digital divide is also influenced by the choices of managers of medical services [49]. Innovations chosen earlier were not necessarily developed with a consideration of people at risk of SHI and may pose problems for these individuals [49]. Managers also have the role of evaluating a potential eHealth tool with respect to its universal (or significant) access.

The digital divide may also be accentuated in the stage before the utilization of an eHealth tool, that is, the search for assistance, information, or services (help-seeking). Indeed, people who have less of a tendency to seek information and to use services are those most at risk of SHI [41,46,47,50]. However, a number of them will still seek information in their local community [51] or when they are particularly interested in a subject [52].

Finally, it appears that the digital divide is more a continuum than a dichotomous concept [53]. The consequences of the digital divide on the health of individuals have been recognized by the United States since the turn of the century. It is now a matter of justice in health [42], since the digital divide in eHealth is a significant barrier that serves to accentuate SHI [49].

### Characteristics of People at Risk of Experiencing a Situation of Social Inequality in Health

SHI and the digital divide generally affect the same individuals [37,54]. eHealth tools are primarily developed for people with good digital skills and Internet access [39,49]. Meanwhile, most nonusers of the Internet are older people or those with low income. Thus, inequalities are accentuated for these groups [42]. An effective design of an eHealth tool for one group could bring about negative and unforeseen consequences for another group with different characteristics (physical, cognitive, or cultural) [16]. Ethnicity (48) and low income (47) are the most common characteristics. Next comes a low level of education (34), age (26), a low literacy level (18), gender (14), rurality (11), incapacities (8), psychological distress (1), homelessness (1), and sexual orientation (1). According to Feng’s study [44], groups identified as particularly disadvantaged in the utilization of social networks are low-income individuals, those with little education or literacy problems, the unemployed, the aged, the handicapped, women, and the people of ethnic origin. However, since Feng [44] used the correction of Bonferroni in his analysis and, thus, chose a more conservative stance, it is possible that certain links were not brought to light (see Table 1).

**Table 1:** Characteristics of people at risk of experiencing a situation of social inequality in health.

This review (n)	Feng [44]
Ethnicity (48)	Ethnicity
Low income (47)	Low income
Low level of education (34)	Low level of education
Age (26)	Age
Low literacy level (18)	Low literacy level
Gender (14)	Gender
Rurality (11)	
Incapacities (8)	Incapacities
Psychological distress (1)	
Homelessness (1)	
Sexual orientation (1)	

In Canada, it appears that income is the factor with the greatest impact on Internet access, more than other factors such as education level, geographic location, gender, and age [55]. However, research on racial and ethnic health inequalities has demonstrated that SHI may persist despite the inclusion of measures related to socioeconomic status [56]. Thus, people with average family income could be at risk of SHI if they belong to another ethnic community.

Although older individuals are the group for whom the use of the Internet is growing most rapidly, this is still a group that uses it the least [57]. Also, within this group, certain disparities exist. Seniors from a minority ethnic group, with little education and literacy, aged 75 years older, or with low income are much less likely to use the Internet [57]. The presence of a number of cognitive and psychomotor barriers related to age may make it difficult to use digital technology, and the effort required to master a new technology can then be perceived as greater than any possible benefits [57]. In general, for older people, and even more for those with low income and incapacities, literacy and Internet access are important factors in explaining the digital divide [58]. Finally, in terms of gender, although women have a tendency to use technology less, they still use eHealth more [59,60].

What can be done to lessen the digital divide (primary, secondary, and tertiary)? Four promising strategies for the development of the eHealth tool are highlighted in the analysis of different studies examined in the context of this review.

### Promising Strategies for Development of the eHealth Tool to Reduce Social Health Inequalities

#### Ensuring Universal Access to the eHealth Tool

To guarantee universal access and reduce the digital divide, it is important to clearly understand the systemic barriers, which potential users may confront [42]. An approach centered on the user is recommended, placing the person’s needs, preferences, capacities, values, and goals in the forefront, in particular, when this concerns people at risk of SHI.

To reduce problems of physical access to a computer, the strategies generally proposed are increasing the quantity of computers available in public spaces such as libraries and community centers or providing more personal computers in people’s homes [47,58]. The former solution has the advantage of being more economical. However, it may also constitute a violation of confidentiality, particularly when content related to health is involved [47]. Even if computers are available in libraries, problems with transportation may limit this solution. As for personal computers, Ryan [61] illustrates some very concrete difficulties related to the utilization of an eHealth tool at home. For example, some participants have transportation problems and were unable to get their equipment repaired by the provider who did not make home visits. Another participant burned the motherboard of the personal computer (PC) because the latter was plugged into the same electrical outlet as a kitchen appliance; there were a limited number of outlets in the house. Other participants could not pay for sufficient bandwidth to use the tool. Thus, the importance of access to quality Internet bandwidth should not be underestimated. eHealth tools employ modalities that require a certain performance (in terms of graphics, software, and interfaces), including the downloading of documents for later use or the participation in forums to share with peers [40]. It has also been suggested that hospitals and health services offer free Internet access to their patients [55]. The utilization of mobile devices (tablets and mobile phones) is higher among people at risk of experiencing SHI [18]. This type of technology needs more research, but is still promising in terms of increasing access to eHealth [45,62,63]. Furthermore, a combination of online and offline tools may prove necessary, along with more traditional technologies such as the telephone, printed material, digital versatile disks (DVDs), and printed mail [49,53,57,64].

However, it is not sufficient to provide a tool. It appears that it is necessary to ensure that the future user has the knowledge required for an optimal utilization of the tool on offer. Thus, training and technical assistance are crucial, according to the authors [39,47,57,58,64,65]. In that regard, it is possible to create a support network to bolster users’ skills. For example, volunteers could help older people to learn to use the Internet [40]. Beyond the usage of an eHealth tool, it is also crucial that users be trained to be able to evaluate the quality of sources on the Internet [66]. However, Chu’s study [66] suggests that the attrition rate for this type of training is problematic. Motivation then becomes a critical factor [67].

#### Respecting Users’ Level of Literacy

In designing the tool, the patient’s literacy level and principal language, as well as access and facility of use, must be considered [68]. Thus, audio accompaniment, available in a variety of languages, could compensate for literacy difficulties [66,68]. Certain authors have tested the utilization of modalities of providing health information that demand less in terms of reading skills. They suggest more educational entertainment, using animation or multimedia narrative tools (television programs, video capsules, and so on) [41,47,66].

#### Creating eHealth Tools That Respect the Cultural Characteristics of Future Users

Bacigalupe [49] and McAuley [12] stress that the cultural component in the development of eHealth tools is critical for populations at risk of SHI and, thus, they suggest using targeted strategies (tools specifically designed for these populations), rather than universal strategies (intended for everyone). A failure to consider beliefs, values, and habits of populations or individuals targeted can lessen the value of the tool developed for these individuals [47]. The utilization of photographs representing populations at risk of SHI and a variety of testimonies, the availability of the tool in a number of languages, and focusing on specific needs of this clientele are concrete examples of strategies favoring the consideration of the cultural dimension in eHealth [49,50,63,69].

#### Inviting the Participation of People at Risk of SHI in Developing eHealth Technologies

The active participation of future users and, in particular, people at risk of SHI, in the development of eHealth tools has the potential to reduce inequalities [49]. Involving future users with diverse perspectives, circumstances, capacities, and experiences in the design process increases the chances that the tool will ensure significant (universal) access [42]. Future users have the skills to evaluate, choose, and use eHealth tools and to gain from the experience [42]. Nonetheless, the involvement of low income or poorly educated people, various ethnic groups, as well as those with low literacy levels, still requires specific abilities on the part of the designer to encourage their active participation in designing an eHealth tool [42].

## Discussion

### Principal Findings

This review of the literature had three objectives: (1) identifying characteristics of people at risk of experiencing a situation of SHI; (2) determining the possibilities for action in the development of eHealth tools that avoid increasing SHI; and (3) modeling the process of using an eHealth tool by people at risk of experiencing a situation of SHI.

For the first objective, we saw that a number of sociodemographic characteristics were brought up in various studies to identify or characterize individuals at risk of SHIs (ethnicity, low income, low level of education, age, low literacy level, gender, rurality, incapacities, psychological distress, homelessness, and sexual orientation). Now, these characteristics should be analyzed with due caution. On one hand, they could contribute to supporting a discourse based on differences, but they also fail to consider the heterogeneity that one finds within a single population group [70]. Thus, it seems essential to ensure a range of characteristics when recruiting participants for studies on SHIs and eHealth.

For the second and third objectives, the results obtained from this review of the literature show that the digital divide, in its primary, secondary, and tertiary forms is the principal cause of the exacerbation of SHI by eHealth and that it affects those people already at risk of SHI [42,47,49]. Alternative ways of modeling the link between eHealth and SHI exist. Among others, the integrative model of eHealth use suggests that disparities in social structures (eg, the demographic data) are linked to SHI through health literacy, motivation to use eHealth, and the person’s capacity to use this technology. In this model, existing SHI are exacerbated by technologies that require a certain level of literacy, sustained motivation, and digital capacities [41]. Also, the Structural Influence model identifies the importance of communication in the relationship between social determinants and results linked to health [40,71,72]. It suggests that the differences among social groups (including ethnic minorities) in the utilization of channels of communication result in an exacerbation of SHI [72]. These are highly interesting models. However, the goal of these models is not the development of eHealth tools, and certain key elements, such as the cultural component and the importance of involving future users, are absent. Thus, descriptive metasynthesis allows us to respond to the second objective. Individuals characteristics linked to SHI will encounter difficulties during the process of using an eHealth tool. First, it is possible that they will be less inclined to seek health-related information or to use an eHealth tool to improve their health [41,46,50]. In the case where these people do initiate a process of looking for help, they will need physical access to digital technology (a computer, electronic tablet, or mobile phone) and sufficient bandwidth [66,61,73]. Then, they need to draw upon their capacities to use the technology. Probably they will lack confidence in their abilities or in the technology and will interrupt the process [11,12,73]. However, if they persevere, they will require a level of health literacy sufficient to understand what the eHealth tool is able to offer them and a capacity to integrate and make use of what has been learned [40,73,74]. Individuals with sufficient income, a high level of education, and adequate digital health literacy will be better able to complete the process and improve their state of health. Thus, it is possible that there are gaps between these groups of individuals in the effective utilization of eHealth tools and, therefore, in the improvement of their health, which will contribute to increasing SHI [72].

**Figure 2:**
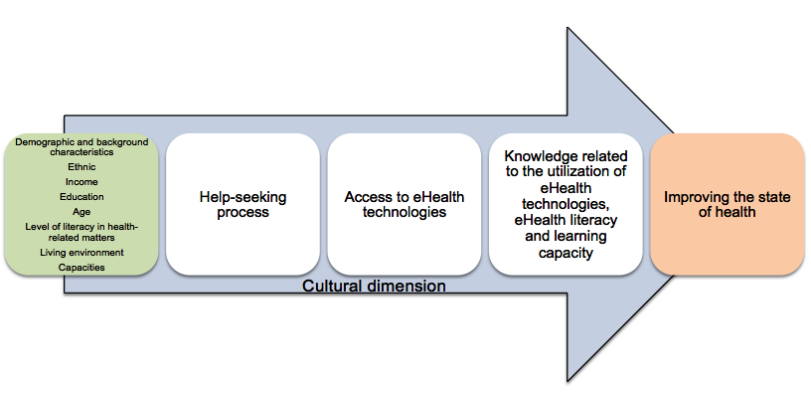
Process of using an eHealth tool.

Nonetheless, if in the designing or adaptation of the tool, the developers consider the future user as a person at risk of SHI [49], design or adapt the tool to respond to the needs of such a user at each stage [42], and integrate the cultural dimension in the process of development [49], it might be possible to reduce the digital divide present in eHealth (Figure 2).

The current increase of technologies in eHealth justifies a reexamination of interventions unlikely to worsen SHI [42,47]. Among other suggestions, it is proposed to target interventions for populations at risk of SHI. Yet, developing an eHealth tool is an undertaking requiring time, energy, and funds. Realistically, developers hope to reach the greatest possible number, and targeted interventions are likely to be rarer. Little participatory research action has been done despite the promising nature of participation of people at risk of SHI in developing eHealth tools to reduce these inequalities. Can we consider developing eHealth tools with the end goal of a universal strategy, but designed to take into account people at risk of SHI and even to involve them in the process? Could we, in developing the tool, question ourselves and question the people at risk of SHI at each stage of the process of using an eHealth tool (Figure 2) and reduce the barriers liable to interrupt the process? Each stage of the process (Figure 2) or conceptualizing category refers to its own field of research. It is difficult, indeed impossible, in the context of this article, to showcase the wealth of knowledge available for each of these concepts. However, the relation between these concepts, more iterative than linear, allows us to envisage a process of coherent codesign, the effect of which might be to reduce SHI.

Although research often raises the potential of eHealth to reduce SHI and offers promising solutions for reducing the digital divide, we agree with Chou [20] that, to date, there are still insufficient empirical studies to prove this definitively, as demonstrated in this review of the literature. Indeed, only three studies examined document the development of an eHealth tool with individuals in a situation of SHI.

### Limitations

The strengths and weaknesses of this study were assessed with the help of AMSTAR [27,33,34]. Although we have attempted to provide a rigorous review of the literature, including a metasynthesis, this review has its limitations. First, in concentrating on a research strategy supported by databases, the gray literature and nontraditional sites of knowledge transfer (eg, the Web) were not included. In addition, we have only used two databases. A limited search can generate a set of studies that are not representative, incomplete results, inadequate selection, and reduced generalization [23]. We have prioritized this choice to ensure greater transparency and reproducibility for this review of the literature. AMSTAR mentions that it is necessary to utilize least two different databases [33]. To avoid biases in the publications, it is recommended not to exclude articles on the basis of year of publication or language. Now, considering that the Internet, social networks, and new technologies have considerably modified the eHealth environment, it was judged sufficient to focus on articles published in the last decade. Furthermore, for reasons of feasibility, the translation of articles was not possible, and free translation software still leaves much to be desired. In accordance with the suggestion of the EPPI group, it was decided to look for articles in all languages initially but, for greater transparency, to exclude articles that are not in languages in which we are fluent [23]. Another limitation of this study is the presence of only a single analyst, which could trigger selection bias. To counter this aspect, often linked to student reality, two supervisors provided support for the writing of this text, and a biostatistician examined the articles from a quantitative perspective. Finally, since the analysis was not based on the quality of studies, the results must be interpreted as possibilities, rather than generalizable facts based on solid data. The rigor of this review stems from the fact that it is systematic (undertaken according to a fixed plan or system or method) and that it is explicit and justified [23]. Nonetheless, because this review does not adopt the same high standards in terms of protection against bias and the quality assessment for the selection of primary research” [75], we called it a “literature review” and not a “systematic review” [75].

### Conclusions

The synthesis of knowledge allowed for (1) a modeling of the process of using an eHealth tool, (2) identifying the actions in eHealth that do not help to reduce SHI, but (3) determining the possibilities for action in the development of tools of eHealth that avoid increasing SHI as well. The massive expansion of technologies in eHealth justifies the study of interventions less likely to exacerbate SHI through the usage of eHealth, and few current empirical studies reveal concrete and effective solutions. Furthermore, very few studies involve future users at risk of SHI. Research is still necessary for eHealth to fulfill its promise to reduce SHI.

## Appendix

Multimedia Appendix 1 Included articles.

Multimedia Appendix 2 Quality of included articles.

## Abbreviations

AMSTAR: assessment of multiple systematic reviews
CINAHL: Cumulative Index to Nursing and Allied Health Literature
DVD: digital versatile disk
EPPI: Evidence for Policy and Practice of Information of the Institute of Education at the University of London
HIV: human immunodeficiency virus
PC: personal computer
SHI: social health inequalities
