# Supplementary material for: Social Health Inequalities and eHealth: A Literature Review With Qualitative Synthesis of Theoretical and Empirical Studies
Source: J Med Internet Res. 2017 Apr 27;19(4):e136. doi: 10.2196/jmir.6731 (PMC5427250; doi:10.2196/jmir.6731)
Supplement: Multimedia Appendix 1 [file jmir_v19i4e136_app1.pdf]

Multimedia Appendix 1. Included articles

N/A. Not Applicable
N/M. Not mentioned

First Author	Study goal	Type of technology	Participants	Design	Key findings
Anthony [56]	To examine how socio-demographic factors, particularly race/ethnicity and gender, as well as health status and health care context are associated with views of electronic health records.	Electronic personal health records	Sample (n = 3,320) is composed of respondents with Internet access who reported making one non-emergency room visits in the 12 months prior to the survey.	Quantitative study based on Health Information National Trends Survey (HINTS)	Racial and ethnic minorities were more likely to believe that an EHR was very important for themselves than whites. The respondents' average level of psychological distress is positively associated with this likelihood. Women to have lower odds of perceiving EHRs to be very important for their providers.
Apter [68]	To examine the opportunity for health organizations and providers to use health information technology to match health information demand to patients' skills and backgrounds and the risk that access to health	Electronic personal health records	10 adults with moderate or severe asthma	Qualitative Study with focus group.	They founded that patients value the information available in a portal. All participants accomplished with ease the seven tasks link with portal use.

	information technology will not be equally available to all patient groups.				
Atkinson [65]	To conduct community needs and assets assessments in 5 rural Maryland counties prior to implementing an Internet-based intervention for low-income mothers	Internet	Stakeholders (n = 58) were community leaders and personnel from 5 targeted Maryland counties working in the areas of community health	Qualitative study with interviews	Stakeholders in all counties were supportive of the proposed health education intervention, feeling it could provide another way to increase residents' health knowledge and awareness of resources.
Atkinson [76]	To conduct formative research to design and develop an Internet-based health education intervention promoting nutrition and physical activity among rural mothers with limited resources using an iterative user-centered approach	Internet	Limited-income adult females	Participatory research action with User-centered design	Women with limited incomes were enthusiastic about a website providing nutrition and physical activity information targeted to their incomes and tailored to their personal goals and needs.
Bacigalupe [49]	To advance in the development of a model that assess the potential of social media tools to address perennial health care quality and access	E-Health tools (Internet, Social networks, mobile health applications, Electronic	N/A	Qualitative study with online ethnography, case analysis, and	E-Health tools are often designed and aimed at patients who have better digital and health care access and not

	inequities in the United States	personal health records)		consultation with experts	deliberately built to reach the most vulnerable populations. As a result, the development of e-Health tools may maintain or even widen health care inequity.
Baur [42]	To analyze the factors that creates and sustains e-Health disparities.	E-Health tools (Internet, Social networks, mobile health applications, Electronic personal health records)	N/A	Theoretical article	The paper presented data and research findings that show existing disparities in income, education, literacy, and health status are mirrored and possibly reinforced by differences in access to the Internet.
Beacom [47]	To understand the communication of health information among disadvantaged populations.	Internet	N/A	Theoretical article	Among disadvantaged populations, information no seeking and avoiding behaviours, coupled with the effects of the knowledge gap, create information disparities that may in turn contribute to health disparities.

Bell [77]	To examine the social factors driving communication inequalities.	Internet	58 Women of low and high socioeconomic status	Qualitative study with interviews	Women of high SES have access to support groups, physicians, and the Internet, whereas women of low SES do not discuss their health problems with their peers, and lack access to and distrust physicians.
Bennett [62]	To raise the question of whether the 2-1-1 system can be leverage as a platform to connect callers to health-related programs and services.	Mobile technology	N/A	Theoretical article	The author proposes blending 2-1-1's core mission of connection with e-Health offerings might make it a little easier to reach socioeconomically disadvantaged populations with efficacious e-Health interventions.
Bhandari [50]	To examine how consumers' online health information seeking behavior is related to different types of healthcare access barriers reported by individuals.	Internet, chat group and e-mail	27 210 adults	Quantitative study based on 2009 National Health Interview Survey.	The internet may offer a low-cost source of health information and could help meet the heightened demand for health-related information among those facing access

					barriers to care.
Bodie [41]	To explicate a theoretical framework capable of aiding his understanding of how e-Health literacy and health disparities are related.	Internet	N/A	Theoretical article	The author proposes that structural inequities reinforce themselves and continue to contribute to healthcare disparities through the differential distribution of technologies that simultaneously enhance and impede literacy, motivation, and ability of different groups (and individuals) in the population.
Breitenstein [45]	To provide an initial evaluation of the feasibility and acceptability of a Web-based delivery prototype of evidence based parent program with a group of low-income, ethnic minority parents.	Applications for tablets and smartphones	9 parents	Mixed methods study based on self-report measures, checklist, survey and focus group.	The Web-based delivery adaptation was feasible and useful for low - income urban parents.
Brouwer [78]	To examine how many people were reached with an implemented multi-risk behavior intervention	Internet	285 146 visitors of website	Quantitative study based on server statistics	It is concluded that a heart-healthy computer-tailored Internet program can reach substantial

					numbers of people, but additional research is needed to develop promotional strategies that reach the high-risk population, i.e. men, older and lower educated persons.
Carlson [54]	To understand the role of Internet and public libraries in the health of African Americans in Charleston and Georgetown counties in South Carolina.	Internet	Nine member planning committee from the faith community, public libraries, grassroot diabetes advocacy groups, rural community centers, and information technology community.	Participatory research action with mixed methods	Older (older than 60) and less educated (fewer than 12 years of education) African Americans in Charleston and Georgetown counties lack skills to access Internet and library services and suffer disparities in health information.
Chilukuri [63]	To evaluate differences in the use of ICT between racial and ethnic groups and by English language proficiency.	Information and communication technology (ICT)	246 women who were aged 18 years or older and pregnant or within 1 year of delivery.	Quantitative study based on self-administered survey	Mobile phones are widely available for the delivery of health interventions to low-income, racially diverse pregnant and postpartum women, but disparities in Internet use and SMS

					text messaging exist. Interventions or programs requiring Web-based apps may have lower uptake unless alternatives are available, such as those adapted for limited English proficiency populations.
Choi [58]	To examine Internet use patterns, reasons for discontinued use, e-Health literacy, and attitudes toward computer/Internet use among low-income homebound individuals aged 60 and older in comparison to their younger counterparts—homebound adults under age 60.	Internet	980 recipients of home-delivered meals in central Texas (78% were age 60 years and older and 22% under age 60).	Quantitative study based on survey	It shows very low rates of Internet use compared to the US population, either due to lack of exposure to computer/Internet technology; lack of financial resources to obtain computers and technology; or medical conditions, disabilities, and associated pain that restrict use.
Chou [79]	To identify the sociodemographic and health-related factors associated with current adult social media users in the United States.	Social networks	5078 adults	Quantitative study based on 2007 Health Information National Trends Study (HINTS)	Recent growth of social media is not uniformly distributed across age groups; therefore, health communication programs utilizing

					social media must first consider the age of the targeted population to help ensure that messages reach the intended audience. While racial/ethnic and health status–related disparities exist in Internet access, among those with Internet access, these characteristics do not affect social media use.
Chou [20]	To examine the impact of Web 2.0 and social media on health promotion and their utility	Internet and Social networks	N/A	Literature review	The scarcity of empirical evidence points to the need for more interventions with participatory and user generated features. Innovative study designs and measurement methods are needed to understand the communication landscape and to critically assess intervention effectiveness. To address health

					disparities, interventions must consider accessibility for vulnerable populations
Chu [66]	To narrow the digital divide and unequal access to technology among older adults in lower socioeconomic communities by designing an intervention that would assist older adults in developing skills and competence in online consumer health information retrieval and evaluation.	Internet	Older adults, sixty-five years and above, enrolled at congregate meal sites of the YWCA	Quantitative study based on randomized control group.	Findings showed a reduction in computer anxiety and increases in computer confidence and computer self-efficacy in retrieving and evaluating online health information ($P < 0.001$).
Dart [80]	To determine the current utilisation, importance, trust and future preference for the Internet as a source of health information in three different socioeconomic groups.	Internet	262 adults	Quantitative study based on survey questionnaire	The Internet was a much more important source of health information for the university sample. The use of online health information and he importance ascribed to the Internet as a source of health information was related to home Internet access and the frequency if

					Internet use in all three populations.
Dart [81]	To determine whether the community's attitudes to components of a community e-Health strategy differ across three different socioeconomic groups.	Internet	Low socioeconomic community (n = 262), a mid-high socioeconomic community (n = 256) and at a local university (n = 200).	Quantitative study based on survey questionnaire	Different communities have different information demands but there is a strong demand for information, which empowers community members to take control of their own health and become active participants in their health care.
Dorstyn [17]	To summarise the available evidence and determine the effectiveness of telecounselling for adults of a minority racial or ethnic group	Telecounselling	N/A	Literature review	Significant short-term treatment effects were associated with telephone- and Internet-mediated services, including moderate to large improvements across measures of depression, anxiety, quality of life and psychosocial functioning reported. Longer-term treatment effects were also reported, although these results were based on

					very limited data
Dudas [82]	To assess pediatric caregiver access to and attitudes toward the use of electronic communication modalities to communicate with health care providers in an urban pediatric primary care clinic.	Emails	300 caregiver-child dyads with children aged from birth to 21 years presenting for care at an urban pediatric primary care clinic in Baltimore, Maryland	Quantitative study based on survey questionnaire	Of the 229 caregivers who completed the survey (91.2% response rate), 171 (74.6%) reported that they use email to communicate with others. Of the email users, 145 respondents (86.3%) stated that they would like to email doctors, although only 18 (10.7%) actually do so. Among email users, African-American caregivers were much less likely to support the expanded use of email communication with health care providers (adjusted OR 0.34, 95% CI 0.14-0.82) as were those with annual incomes less than US $30,000 (adjusted OR 0.26, 95% CI 0.09-0.74)
Eddens [69]	To explores the availability of online cancer survivor stories by	Internet	N/A	Quantitative study based on websites	All racial minority groups combined accounted for 9.8% of

	race and ethnicity of the survivor.			analysis	online cancer survivor stories, despite making up at least 16.3% of prevalent cancer cases. Also notably underrepresented were stories from people of Hispanic ethnicity (4.1%), men (35.7%), survivors of colon cancer (3.5%), and older adults.
Eysenbach [83]	To discuss about e-Health and human development	E-Health tools	N/A	Theoretical article	The author proposes a reflection about how information technology can make the world a more equitable and better place.
Feng [44]	To examine the potential demographic factors that influence SNS use to determine whether there are access inequalities in the social media environment.	Social networks	2 253 adults	Quantitative study based on Pew Internet's Health Tracking Survey in 2006, 2008, and 2010.	The socioeconomic and demographic factors that contributed to the disparities in social networking site use could also lead to disparities in seeking health information online. People are more likely to seek heath-related information online if

					they or their close family or friends have a chronic disease situation.
Fowles [84]	To determine perceptions of accuracy and ease of use of PDA-based dietary records in a sample of low-income pregnant women and the feasibility of using PDA-based dietary record software in this population.	Electronic personal health records	10 low income mothers	Quantitative study based on two food recording methods compared.	The personal digital assistant was significantly easier to use compared to the 24-hour recall, and no significant differences in ease of remembering food intake between these methods was noted. Most women liked the PDA and felt it was more accurate than 24-hour recalls. Results showed no significant difference in mean HEI scores between food records from 24-hour recall to PDA.
Geraghty [67]	To examine and identify predictors of attrition that are likely to inform the development of future large-scale global interventions.	Internet	17 430 adult smokers	Quantitative study	Significant attrition predictors in the automated follow-up cohort included higher levels of nicotine dependency, lower education, lower quitting confidence and

					receiving more contact emails. Participants' younger age was the sole predictor of attrition in the live follow-up cohort.
Gibbons [16]	To identify those best practices, guidance, and standards that could help identify and/or address embedded design assumptions in HIT that could negatively impact patient safety, particularly for non-majority HIT user populations.	Health information technology	N/A	Literature review	Design principles that may help identify and address embedded HIT design assumptions are available in the existing literature.
Gibbons [85]	To obtain guidance regarding the development of a research and action agenda that will use health IT and other quality improvement strategies to reduce disparities for priority populations in underresourced settings.	Health information technology	N\M	Qualitative study based on expert workshop	Several overarching themes, key recommendations, and research topics emerged across five general categories of (a) the health care delivery setting, (b) research and evaluation methodologies, (c) patients and target populations, (d) technology applications and platforms, and (e)

					providers and clinicians.
Gilmour [55]	To present that on-line health information particularly benefits the already privileged in terms of health care; well-educated people with access to economic resources.	Internet	N/A	Theoretical article	On-line health information is a powerful medium for quick and dynamic knowledge distribution. The challenge for nurses and other health professionals is directing that knowledge to the groups most disadvantaged in the current health care systems, with an agenda of reducing inequalities in access to health information.
Gordon [57]	To developed specifications for a suite of e-Health applications to improve the quality of perinatal mental health care.	Applications for tablets and smartphones (m)	Low-income women with a history of antenatal depression, their prenatal providers, mental health specialists, an app developer, and researchers.	Participatory research action based on rapid prototyping	Three apps were developed by the group: an app to support high-risk women after discharge from hospital, a screening tool for depression, and a patient decision aid for supporting treatment choice.
Gordon [86]	To assess the extent to	Applications for	Seniors aged	Quantitative	Health plans,

	which race/ethnic and age-related e-Health digital divides exist among the racially and ethnically diverse seniors of Kaiser Permanente Northern California and what might be driving the divides that are observed.	tablets and smartphones (m)	65-79 years	approach based on patient portal account status and mailed survey.	government agencies, and other organizations that serve diverse groups of seniors should include social determinants such as race/ethnicity and age when monitoring trends in e-Health to ensure that e-Health disparities do not induce greater health status and health care disparities between more privileged and less privileged groups.
Huxley [19]	To understand how the intervention (digital clinical communication) works in specific contexts (groups marginalised from general practice access) with what outcome (access to clinical care in general practice).	Digital communication	N/A	Literature review	Digital communication technology offers increased opportunities for marginalised groups to access health care. However, it cannot remove all barriers to care for these groups.
Jones [73]	To develop a patient-completed tool giving patients' perceptions of their opportunity that could be combined with	E-Health tools	344 adults	Quantitative study based on survey	The Patient e-Health Readiness Questionnaire (PERQ) appears acceptable for

	their personal abilities, their support networks, and economic barriers.				participants in British studies. The scores produced appear valid and will enable assessment of the effectiveness of interventions to improve patient e-Health readiness and reduce e-Health inequalities.
Kerr [87]	To explore the potential of a web-based intervention for reaching a large number of patients, including those in disadvantaged groups, by examining: (1) the participation level in a study evaluating a web-based intervention for coronary heart disease (CHD), and (2) the level of use of the intervention by the participants.	Internet	168 with coronary heart disease	Mixed methods Study based on questionnaire and interviews.	The availability of a web-based intervention, with support for use at home or through public Internet services, did not result in a large number or all types of patients with CHD using the intervention for self-management support. The effectiveness of web-based interventions for patients with chronic diseases remains a significant challenge.
Kontos [13]	To employ an up-to-date, comprehensive examination of e-Health	Social networks (2010)	2368 adult Internet users	Quantitative study based on National Cancer	Lower SES, older, and male online US adults were less likely to

	use by sociodemographic factors to illustrate potential profiles of disparities across a number of communication domains			Institute's 2012 Health Information National Trends Survey (HINTS)	engage in a number of e-Health activities compared to their counterparts
Kontos [72]	To identify potential disparities in adult SNS use by race/ethnicity and socioeconomic position to determine if there are communication inequalities in this segment of the social media environment and to examine the association between SNS use and psychological well being to determine if the beneficial impact of SNS use observed among teens holds true among an adult population.	Social networks	3582 adults	Quantitative study based on National Cancer Institute's 2007 Health Information National Trends Survey (HINTS).	The absence of inequalities in adult SNS use across race/ethnicity and class offers some support for the continued use of social media to promote public health efforts. However, issues such as the persisting digital divide and potential deleterious effects of SNS use on psychological well being need to be addressed.
Kontos [40]	To investigate how members of lower SEP groups, people who currently have limited access, use and experience the Internet.	Internet	12 low-SEP urban individuals with no or limited computer and Internet experience	Qualitative study based on interviews	Major barriers to Internet use included time constraints and family conflict over computer usage. The monthly training classes and technical assistance components of the

					intervention surfaced as the most important facilitators to computer and Internet use. The concept of received social support from other study members, such as assistance with computer-related questions, also emerged as an important facilitator to overall computer usage.
Kukafka [88]	To describe the steps taken by the HHPC to develop a community-specific health web portal.	Internet	646 adults (survey), N/M (focus group)	Participatory research action based on survey and focus group.	Sentiments of distrust in medical institutions, and the desire for community specific content and resources were among the needs emanating from our data analysis.
Lee [52]	To examine whether there are any systematic differences in cancer information seeking between high- and low-socioeconomic status (SES, hereafter) cancer patients, and (2) what factors moderate the	Internet	2013 patients with breast, prostate, and colon cancer	Quantitative study based on survey	Education was more strongly associated with Internet use than with the use of other sources regardless of topics. Also, when information was sought from mass

	effect of SES on cancer information seeking among cancer patients.				media, education had a greater association with treatment information seeking than with quality-of-life information seeking. Preference for active participation in treatment decision making, however, did not moderate the effect of education on treatment information seeking.
Lindsay [37]	To examine whether having access to a purpose-built health portal might make a difference to patients in how they self-manage their heart disease.	Internet	108 men and women aged 50–74 from coronary heart disease registries.	Mixed methods based on randomized controlled trial and discussion forums.	The experimental group changed their diet significantly. They reported eating ‘bad foods’ (such as chips, sweets, crisps, fried foods, ready meals and cakes/biscuits) significantly less often compared to the controls.
Liszka [89]	To determine the extent of Internet access and online health seeking and the feasibility of implementing Internet services for an urban,	Internet	300 patients	Quantitative study based on survey	Seventy-seven percent of respondents, characterized by low socioeconomic status, low education level,

	residency-based practice.				and high minority percentages, had accessed the Internet at least once, 79 percent had used the Internet to find health-related information, 73 percent used the online information to make a health-related decision, and 50 percent shared the information with their provider.
Lorence [90]	To examine whether education levels of Internet health information seekers are correlated with distribution patterns and diffusion trends in access to computers, Internet, and online health information.	Internet	4227 respondents	Quantitative study based on two datasets of the Pew Internet & American Life Project from March 2000 and December 2002	Recent policy initiatives under national technology access and other programs have demonstrated little effect in narrowing the digital divide for low-education users of web-based technologies.
Lustria [8]	To examines relationships of health consumers' use of web resources/technologies for health information seeking, personal health information management	E-Health tools	3582 respondents	Quantitative study based on 2007 Health Information National Trends Survey (HINTS)	Patterns indicate early evidence of a narrowing divide in e-Health technology use across population groups as a result of the narrowing divide

	and patient-provider communication.				in Internet access and computer ownership warrants further exploration and need to explore differences in the use of e-Health tools by medically underserved and disadvantaged groups.
Malone [91]	To examine the impact of psychosocial factors upon local 'health literacy' as related to information access, usability, usefulness and relevance and both 'digital' and 'preference' divides.	Internet	Families with children under five years of age and living in five socially, economically and culturally disparate local authority (LA) wards	Mixed methods study based on questionnaires, focus group and interviews.	Multiple layers of influence upon parental health information seeking emerged and revealed a non-digital second divide, which was independent of computer ownership and home Internet access. This divide was based on preference for use of certain health information sources, which might be either 'online' or 'offline'. A spatial patterning of both digital and preferential divides was identified with an association between each of

					these and features of the physical, social, cultural and psychosocial environment, one of which was perceived access to primary health care.
Massey [53]	To describe population characteristics and sources of health information among U.S. adults who do not use the Internet.	Internet	1722 respondents	Quantitative study based on Health Information National Trends Survey	Older adults, minority populations, and individuals with low educational attainment represent a growing percentage of respondents who have looked for health information but have never used the Internet, highlighting trends in digital information disparities.
McAuley [12]	To summarize the so-called 'digital divide', consider how it may negatively impact on health and health inequalities, and suggest ways in which such impacts can be mitigated against	E-Health tools	N/A	Theoretical article	Digital health interventions offer a potentially important new development for prevention and treatment provided they are proven to be effective and cost effective. The use of digital technology can be seen both as an

					indicator of socio-economic status and a facilitator for better health outcomes. However, issues of equity require more appropriate recognition as digital health interventions may potentially widen health inequalities rather than narrow them.
McInnes [21]	To synthesize what is known about access to and use of information technologies in homeless populations.	E-Health tools	N/A	Literature review	Many homeless persons had access to information technologies, suggesting possible health benefits to developing programs that link homeless persons to health care through mobile phones and the Internet.
Miller [60]	To examine the degree to which health care consumers seek health information through conventional, face-to-face consultation, telemedicine, or digital technology and	E-Health tools	1,428 adults	Quantitative study based on survey	Few people are using digital technology to get information, communicate with health personnel, or make online medical purchases. Less well educated, lower-

	comparing the relationship between these means of communication and demographic factors and health care perceptions.				income individuals living in rural areas tend to use the health care Internet less than others.
Montague [18]	To examine ways in which technology is being used by historically underserved populations in order to decrease the health disparity through facilitating or improving health care access and health and wellness outcomes.	E-Health tools	N/A	Literature review	Technology can be used to positively affect the health of historically underserved populations, the technology must be tailored toward the intended population, as personally relevant and contextually situated health technology is more likely than broader technology to create behavior changes. Social media, cell phones, and videotapes are types of technology that should be used more often in the future.
Morey [39]	To discuss the digital divide	Internet	N/A	Theoretical article	Some view the Internet as one solution to eliminating health

					disparities, although barriers such as education, income and cost, age, location, literacy, and content contribute to a persistent digital divide. Many solutions to this problem have been proposed, but the situation still warrants further study before e-Health strategies and interventions can be effectively implemented to a wider community.
Munoz [92]	To explore the use of evidence-based Internet interventions for health problems could contribute to the reduction of one aspect of health disparities, namely, having inadequate or no access to evidence-based interventions.	Internet	N/A	Theoretical article	To reduce health disparities worldwide, the international community should develop a system to provide evidence-based Internet interventions at no cost to the users. To launch this process, funding agencies and globally minded foundations or

					corporations would provide ongoing support to host and maintain automated self-help Internet interventions. The number of people who could benefit from such evidence-based Internet interventions would be massive. The return on investment on Internet interventions that can be used again and again is much higher than from provision of consumable interventions whose therapeutic power is spent after one use.
Neuenschwander [38]	To determine the impact of a web-based nutrition education program for low-income adults on changes in nutrition behaviors after three nutrition education lessons.	Internet	123 Low-income adults	Quantitative study based on randomized block equivalence trial	Most nutrition-related behavior outcomes (eg, fruit, vegetable, whole-grain intake, Nutrition Facts label use, breakfast, and meal-planning frequency) improved significantly ($P<0.05$) from pre to post

					within both groups, meaning that each intervention was effective.
Neuenschwander, [93]	To investigate the access to different types of technologies, such as the Internet, among Indiana's low-income population, to ascertain whether Web-based nutrition education was an option for a low-income population.	Internet	1620 low income adults	Quantitative study based on questionnaire	The results of this study provide evidence that using Internet-based nutrition education in a low-income population is a viable and possibly cost-effective option.
Newman [94]	To examine ICT use, perceived barriers and facilitators, and preferences for provider contact in lower income and disadvantaged groups in South Australia.	Internet	80 low income adults	Qualitative study based on focus groups	Barriers and facilitators included English literacy (including for native speakers), technological literacy, education, income, housing situation, social connection, health status, employment status, and trust. Many people gained ICT skills by trial and error or help from friends, and only a few from formal programs, resulting in varied skills.
Nolke [95]	To generate a profile of	Internet	2000	Quantitative	In order to achieve

	users and non-users of online health information, in order to identify discriminating factors between users and non-users.		respondents	study based on Survey	equity in health, health-related Internet use by the socially deprived should be promoted through measures to increase their level of e-Health literacy
Paul [64]	To assess levels of Internet access, likelihood of using various sources of information or support, and sociodemographic characteristics related to high internet access among support persons of haematological cancer patients.	Internet	268 patients	Quantitative study based on survey	Support persons who potentially are more vulnerable due to age and lower education are the least likely to use Internet-based options. Consequently, these groups may require alternatives, including face-to-face or print-based information and support.
Piette [22]	To highlight gaps in our knowledge of the benefits of e-Health and identify areas of potentially useful future research on e-Health.	E-Health tools	N/A	Literature review (Scoping review)	Although large programmes for e-Health implementation and research are being conducted in many low- and middle-income countries, more information on the impacts of e-Health on outcomes

					and costs in these settings is still needed.
Reinwand [6]	To identify personal and socioeconomic characteristics associated with recommended e-Health intervention use.	Internet	1638 participants	Quantitative study based on randomized controlled trial	E-Health interventions were used differently by subgroups. The more frequent as-recommended intervention use by unemployed, older, and ill participants may be an indication that these e-Health interventions are attractive to people with a greater need for health care information.
Ryan [61]	To determine the feasibility of this Internet-based intervention that targets very low-income minority patients who are not likely to engage in diabetes self-management activities and to determine whether utilization contributes to increased participation in diabetes self-management and improved diabetes outcomes.	Electronic personal health records	21 patients	Quantitative study based on feasibility trial	To study the demographics of users who participate in health-related Web-based social outlets to identify possible links to health care disparities.

Sadah [96]	To study the demographics of users who participate in health-related Web-based social outlets to identify possible links to health care disparities.	Social networks	N/M	Quantitative study based on key statistics from three different types of health-related social outlets	Female users dominate drug review websites and health Web forums. The participants of health-related social outlets are generally older with the exception of the 65+ years bracket. Blacks are underrepresented in health-related social networks. Users in areas with better access to health care participate more in Web-based health-related social outlets. The writing level of users in health-related social outlets is significantly lower than the reading level of the population.
Sarkar [74]	To examine patient use patterns of an innovative Internet-based patient portal within a well characterized, large, diverse cohort of adult, medically insured patients with diabetes cared for in	Electronic personal health records	14 102 patients	Quantitative study based on electronic member database.	Those most at risk for poor diabetes outcomes may fall further behind as health systems increasingly rely on the Internet and limit current modes of

	an integrated delivery system in the USA.				access and communication.
Schmeida [97]	To explore the disparities in accessing the Medicare and Medicaid programs, and other public health information through the Internet that limit the effectiveness of telehealth as a policy tool.	Internet	2928 adults	Quantitative study based on survey	Some disparities are narrowing as the elderly and poor in need of access to public health insurance are searching for it online. However, people without Internet access and experience (perhaps the oldest and poorest) remains disadvantaged with respect to accessing critical information that can link them to needed health care services.
Vanderbilt [98]	To determine if the computer-based educational modules related to preterm birth health literacy and health disparity can effectively increase health knowledge of our participants in targeted underserved communities within the Richmond-metro area.	Internet	140 participants	Quantitative study based on pre-post survey	The participants demonstrated an increase in their knowledge in health literacy and preterm birth.

Viswanath [11]	To shed light on the Internet use and browsing patterns among the urban poor in New England.	Internet	312 participants	Quantitative study based on survey and Internet use tracking software	Internet use increased among Intervention participants, with most of their time spent on social and participatory media sites or Internet portals. Purposive searching for health information was low among all participants. Social networking sites were frequently visited, with three sites enjoying similar popularity among all groups.
Viswanath [99]	To investigate the relationships that race, ethnicity, language, and social class have with the use of health communications including cancer information seeking, attention to health information in the mass media, and trust of cancer information from these media.	Communication technologies	5187 U.S. adults	Quantitative study based on survey	Health media use is patterned by race, ethnicity, language and social class. Providing greater access to and enhancing the quality of health media by taking into account factors associated with social determinants may contribute to addressing social

					disparities in health.
Viswanath [71]	To discuss about health disparities et e-Health	Communication technologies	N/A	Theoretical article	Systematic and focused approach to research and application of findings in policy and practice is needed to ensure that exciting and promising developments in e-Health benefit all members of society.
Wang [100]	To examine Internet use among people with limited ability to travel.	Internet	44 507 respondents	Quantitative study based on 2001 National Household Travel Survey (NHTS)	Digital divide exists between urban and rural residents. Internet use and frequency was lower among those reporting a medical condition than among those without a condition. African Americans and Hispanics were still less likely to use the Internet, and to use it less often, than whites.
West [101]	To investigate the accessibility, privacy, and security of public e-Health focusing on the health department websites	Internet	N/A	Qualitative study based on content analysis of websites	Although progress has been made at improving the accessibility and confidentiality of

	maintained by the 50 state governments in the United States.				health department electronic resources, there remains much work to be done to ensure quality access for all Americans in the area of public e-Health.
Yamin [102]	To compare the demographic characteristics, including age, race/ ethnicity, and socioeconomic status (SES), of individuals who registered to use the PHR (ie, adopters) with those who saw a physician offering a PHR but did not register (ie, nonadopters).	Electronic personal health records	75 056 patients	Quantitative study based on analysis of the use of personal health records.	Despite increasing Internet availability, racial/ethnic minority patients adopted a PHR less frequently than white patients, and patients with the lowest annual income adopted a PHR less often than those with higher incomes. Among adopters, however, income did not have an effect on PHR use.
Zach [47]	To examine the patterns of Internet access and use by the patient population at an urban health centre in a medically underserved area.	Internet	53 patients	Quantitative study based on interviews	Lack of access to the Internet in itself is not the primary barrier to seeking health information in this population and that the digital divide exists not at the level of information access

					but rather at the level of information use.
Zhao [48]	To examine the relationship between parental education and children's online health information seeking in the context of the on-going digital divide debate.	Internet	12 969 respondents	Quantitative study based on the Pew Internet and American Life Project for the US 2004, 2006	Teens of low education parents are either as likely as or even more likely than teens of high education parents to seek online health information.

*The references that follow are consistents with the references of the article.

6. Reinwand DA, Schulz DN, Crutzen R, Kremers SP, de Vries H. Who Follows eHealth Interventions as Recommended? A Study of Participants' Personal Characteristics From the Experimental Arm of a Randomized Controlled Trial. J Med Internet Res 2015;17(5):e115. PMID: 25963607
8. Lustria MLA, Smith SA, Hinnant CC. Exploring digital divides: an examination of eHealth technology use in health information seeking, communication and personal health information management in the USA. Health Informatics J 2011 Sep;17(3):224–43. PMID: 21937464
11. Viswanath K, McCloud R, Minsky S, Puleo E, Kontos E, Bigman-Galimore C…Emmons, KM. Internet use, browsing, and the urban poor: implications for cancer control. J Natl Cancer Inst Monographs 2013 Dec;2013(47):199–205. PMID: 24395992
12. McAuley A. Digital health interventions: widening access or widening inequalities? Public Health 2014 Dec;128(12):1118–20. PMID: 25458115
13. Kontos E, Blake KD, Chou W-YS, Prestin A. Predictors of eHealth usage: insights on the digital divide from the Health Information National Trends Survey 2012. J Med Internet Res 2014;16(7):e172. PMID: 25048379
16. Gibbons MC, Lowry SZ, Patterson ES. Applying Human Factors Principles to Mitigate Usability Issues Related to Embedded Assumptions in Health Information Technology Design. JMIR Hum Factors 2014;1(1):e3. PMID: 27025349
17. Dorstyn DS, Saniotis A, Sobhanian F. A systematic review of telecounselling and its effectiveness in managing depression amongst minority ethnic communities. J Telemed Telecare 2013 Sep;19(6):338–46. PMID: 24163298
18. Montague E, Perchonok J. Health and wellness technology use by historically underserved health consumers: systematic review. Journal of Medical Internet Research 2012 Aug 7;14(4):e78–e78 1p. PMID: 22652979

19. Huxley CJ, Atherton H, Anstey Watkins J, Griffiths F, Watkins JA. Digital communication between clinician and patient and the impact on marginalised groups: a realist review in general practice. British Journal of General Practice 2015;65(641):e813–e821 9p. PMID: 26622034
20. Wen-ying Sylvia Chou, Prestin A, Lyons C, Wen K. Web 2.0 for Health Promotion: Reviewing the Current Evidence. American Journal of Public Health 2013 Jan;103(1):e9–e18 1p. PMID: 23153164
21. McInnes DK, Li AE, Hogan TP. Opportunities for engaging low-income, vulnerable populations in health care: a systematic review of homeless persons' access to and use of information technologies. Am J Public Health 2013 Dec;103 Suppl 2:e11-24. PMID: 24148036
22. Piette JD, Lun KC, Moura LA, Fraser HSF, Mechael PN, Powell J, Khoja, SR. Impacts of e-Health on the outcomes of care in low- and middle-income countries: where do we go from here? Bull World Health Organ 2012 May 1;90(5):365–72. PMID: 22589570
37. Lindsay S, Bellaby P, Smith S, Baker R. Enabling healthy choices: is ICT the highway to health improvement? Health (London) 2008 Jul;12(3):313–31. PMID: 18579630
38. Neuenschwander LM, Abbott A, Mobley AR. Comparison of a web-based vs in-person nutrition education program for low-income adults. J Acad Nutr Diet 2013 Jan;113(1):120–6. PMID: 23092741
39. Morey OT. Digital disparities: the persistent digital divide as related to health information access on the Internet. Journal of Consumer Health on the Internet 2007 Oct;11(4):23–41 19p. http://dx.doi.org/10.1300/J381v11n04_03
40. Kontos EZ, Bennett GG, Viswanath K. Barriers and facilitators to home computer and Internet use among urban novice computer users of low socioeconomic position. J Med Internet Res 2007;9(4):e31. PMID: 17951215
41. Bodie GD, Dutta MJ. Understanding health literacy for strategic health marketing: eHealth literacy, health disparities, and the digital divide. Health Mark Q 2008;25(1–2):175–203. PMID: 18935884
42. Baur C. An analysis of factors underlying e-Health disparities. Camb Q Healthc Ethics 2008;17(4):417–28. PMID: 18724881
44. Feng Y, Xie W. Digital Divide 2.0: The role of social networking sites in seeking health information online from a longitudinal perspective. Journal of Health Communication 2015;20(1):60-68. PMID: 25119019
45. Breitenstein SM, Gross D. Web-based delivery of a preventive parent training intervention: a feasibility study. J Child Adolesc Psychiatr Nurs 2013 May;26(2):149–57. PMID: 23607827
47. Beacom AM, Newman SJ. Communicating health information to disadvantaged populations. Family & Community Health 2010;33(2):152–162 11p. PMID: 20216358
48. Zhao, S. Parental education and children's online health information seeking: beyond the digital divide debate. Soc Sci Med 2009; 69(10): 1501-1505. Doi:10.1016/j.socscimed.2009.08.039

49. Bacigalupe G, Askari SF. E-Health innovations, collaboration, and healthcare disparities: developing criteria for culturally competent evaluation. Fam Syst Health 2013 Sep;31(3):248–63. PMID: 24059273
50. Bhandari N, Shi Y, Jung K. Seeking health information online: does limited healthcare access matter? J Am Med Inform Assoc 2014 Dec;21(6):1113–7. PMID: 24948558
52. Lee C-J, Ramírez AS, Lewis N, Gray SW, Hornik RC. Looking beyond the Internet: examining socioeconomic inequalities in cancer information seeking among cancer patients. Health Commun 2012;27(8):806–17. PMID: 22356137
53. Massey PM. Where Do U.S. Adults Who Do Not Use the Internet Get Health Information? Examining Digital Health Information Disparities From 2008 to 2013. J Health Commun 2016 Jan;21(1):118–24. PMID:26166484
54. Carlson BA, Neal D, Magwood G, Jenkins C, King MG, Hossler CL. A community-based participatory health information needs assessment to help eliminate diabetes information disparities. Health Promotion Practice 2006;7(3):213S–22S 1p. PMID: 16760247
55. Gilmour JA. Reducing disparities in the access and use of Internet health information. A discussion paper. Int J Nurs Stud 2007 Sep;44(7):1270–8. PMID: 16828775
56. Anthony DL, Campos-Castillo C. Do health care users think electronic health records are important for themselves and their providers?: Exploring group differences in a national survey. AMIA Annu Symp Proc 2013:42–9. PMID: 24551321
57. Gordon M, Henderson R, Holmes JH, Wolters MK, Bennett IM. Participatory design of e-Health solutions for women from vulnerable populations with perinatal depression. Journal of the American Medical Informatics Association 2016;23(1):105–109 5p. PMID: 26342219
58. Choi NG, Dinitto DM. The digital divide among low-income homebound older adults: Internet use patterns, eHealth literacy, and attitudes toward computer/Internet use. J Med Internet Res 2013;15(5):e93. PMID: 23639979
60. Miller EA, West DM. Where's the revolution? Digital technology and health care in the Internet age. J Health Polit Policy Law 2009 Apr;34(2):261–84. PMID: 19276318
61. Ryan JG, Schwartz R, Jennings T, Fedders M, Vittoria I. Feasibility of an Internet-based intervention for improving diabetes outcomes among low-income patients with a high risk for poor diabetes outcomes followed in a community clinic. Diabetes Educ 2013 Jun;39(3):365–75. PMID: 23610181
62. Bennett GG. Connecting eHealth with 2-1-1 to reduce health disparities. Am J Prev Med 2012 Dec;43(6 Suppl 5):S509-511. PMID: 23157773
63. Chilukuri N, West M, Henderson JL, Lawson S, Ehsanipoor R, Costigan K…Bennett W. Information and Communication Technology Use Among Low-Income Pregnant and Postpartum Women by Race and Ethnicity: A Cross-Sectional Study. Journal of Medical Internet Research 2015;17(7):1–1 1p. PMID: 26142162
64. Paul CL, Clinton-McHarg T, Lynagh M, Sanson-Fisher RW, Tzelepis F. On-line information and support for supporters and carers of haematological cancer patients: is access an issue? Support Care Cancer 2012 Nov;20(11):2687–95. PMID: 22350593

65. Atkinson NL, Desmond SM, Saperstein SL, Billing AS, Gold RS, Tournas-Hardt A. Assets, challenges, and the potential of technology for nutrition education in rural communities. J Nutr Educ Behav 2010 Dec;42(6):410–6. PMID: 20813589
66. Chu A, Huber J, Mastel-Smith B, Cesario S. "Partnering with seniors for Better Health": computer use and Internet health information retrieval among older adults in a low socioeconomic community. J Med Libr Assoc 2009 Jan;97(1):12–20. PMID: 19159002
67. Geraghty AWA, Torres LD, Leykin Y, Pérez-Stable EJ, Muñoz RF. Understanding attrition from international Internet health interventions: a step towards global eHealth. Health Promot Int 2013 Sep;28(3):442–52. PMID: 22786673
68. Apter AJ. Can patient portals reduce health disparities? A perspective from asthma. Annals of the American Thoracic Society 2014;11(4):608–612 5p. PMID: 24640983
69. Eddens KS, Kreuter MW, Morgan JC, Beatty KE, Jasim SA, Garibay L, Jupka, KA. Disparities by race and ethnicity in cancer survivor stories available on the web. Journal of Medical Internet Research 2009 Oct;11(4):e50–e50 1p. PMID: 19945948
71. Viswanath K, Kreuter MW. Health disparities, communication inequalities, and eHealth. Am J Prev Med 2007 May;32(5 Suppl):S131-133. PMID: 17466818
72. Kontos EZ, Emmons KM, Puleo E, Viswanath K. Communication inequalities and public health implications of adult social networking site use in the United States. J Health Commun 2010;15 Suppl 3:216–35. PMID: 21154095
73. Jones R. Development of a Questionnaire and Cross-Sectional Survey of Patient eHealth Readiness and eHealth Inequalities. Med 2.0 2013; 2(2): e9. DOI: 10.2196/med20.2559
74. Sarkar U., Karter A. J., Liu JY., Adle, NE., Nguyen R., Lopez A., Schillinger D. Social disparities in Internet patient portal use in diabetes : evidence that the digital divide extends beyond access. J Am Med Inform Assoc 2011; 18(3): 318-321. doi: 10.1136/jamia.2010.006015
76. Atkinson NL., Desmond SM., Saperstein SL., Billing AS., Gold RS., Tournas-Hardt A. Assets, challenges, and the potential of technology for nutrition education in rural communities. J Nutr Educ Behav 2009; 42(6): 410-416. Doi:10.1016/j.jneb.2009.09.004
77. Bell AV. "I think about Oprah": social class differences in sources of health information. Qual Health Res 2014; 24(4): 506-516. Doi:10.1177/1049732314524637
78. Brouwer W., Oenema A., Raat H., Crutzen R., de Nooijer J., de Vries NK., Brug J. Characteristics of visitors and revisitors to an Internet-delivered computer-tailored lifestyle intervention implemented for use by the general public. Health Educ Res 2010; 25(4): 585-595. Doi:10.1093/her/cyp063
79. Chou WY., Hunt YM., Beckjord EB., Moser RP., Hesse BW. Social media use in the United States: implications for health communication. J Med Internet Res 2009; 11(4): e48. Doi:10.2196/jmir.1249
80. Dart J. The Internet as a source of health information in three disparate communities. Aust Health Rev 2008; 32(3): 559-569.

81. Dart, JM, Gallois C. Community desires for an online health information strategy. Aust Health Rev 2010; 34(4): 467-476. Doi:10.1071/ah08719
82. Dudas RA., Crocetti M. Pediatric caregiver attitudes toward email communication: survey in an urban primary care setting. J Med Internet Res 2013; 15(10): e228. Doi:10.2196/jmir.2738
83. Eysenbach G. Poverty, human development, and the role of eHealth. J Med Internet Res 2007; 9(4); e34. Doi:10.2196/jmir.9.4.e34
84. Fowles ER., Gentry B. The feasibility of personal digital assistants (PDAs) to collect dietary intake data in low-income pregnant women. J Nutr Educ Behav 2008; 40(6): 374-377. Doi:10.1016/j.jneb.2007.08.015
85. Gibbons MC., Casale CR. Reducing disparities in health care quality: the role of health IT in underresourced settings. Med Care Res Rev 2010; 67(5 Suppl): 155S-162S. Doi:10.1177/1077558710376202
86. Gordon NP., Hornbrook MC. Differences in Access to and Preferences for Using Patient Portals and Other eHealth Technologies Based on Race, Ethnicity, and Age: A Database and Survey Study of Seniors in a Large Health Plan. J Med Internet Res 2016; 18(3): e50. Doi:10.2196/jmir.5105
87. Kerr C., Murray E., Noble L., Morris R., Bottomley C., Stevenson F . . . Nazareth I. The potential of Web-based interventions for heart disease self-management: a mixed methods investigation. J Med Internet Res 2010; 12(4): e56. Doi:10.2196/jmir.1438
88. Kukafka R., Khan SA., Hutchinson C., McFarlane DJ., Li J., Ancker JS., Cohall A. Digital partnerships for health: steps to develop a community-specific health portal aimed at promoting health and well-being. AMIA Annu Symp Proc 2007:428-432.
89. Liszka HA., Steyer TE., Hueston WJ. Virtual Medical Care: How Are Our Patients Using Online Health Information? Journal of Community Health 2006; 31(5):368-378. Doi:10.1007/s10900-006-9019-3
90. Lorence D., Park H. Study of education disparities and health information seeking behavior. Cyberpsychol Behav 2007; 10(1):149-151. Doi:10.1089/cpb.2006.9977
91. Malone M., While A., Roberts J. Parental health information seeking and re-exploration of the 'digital divide'. Prim Health Care Res Dev 2014; 15(2):202-212. Doi:10.1017/S1463423613000194
92. Munoz RF. Using evidence-based Internet interventions to reduce health disparities worldwide. J Med Internet Res 2010; 12(5):e60. Doi:10.2196/jmir.1463
93. Neuenschwander LM., Abbott A., Mobley AR. Assessment of low-income adults' access to technology: implications for nutrition education. J Nutr Educ Behav 2012; 44(1):60-65. Doi:10.1016/j.jneb.2011.01.004
94. Newman L., Biedrzycki K., Baum F. Digital technology use among disadvantaged Australians: implications for equitable consumer participation in digitally-mediated communication and information exchange with health services. Aust Health Rev 2012; 36(2):125-129. Doi:10.1071/AH11042

95. Nolke L., Mensing M., Kramer A., Hornberg C. Sociodemographic and health-(care-)related characteristics of online health information seekers: a cross-sectional German study. BMC Public Health 2015; 15:31. Doi:10.1186/s12889-015-1423-0
96. Sadah SA., Shahbazi M., Wiley MT., Hristidis VA Study of the Demographics of Web-Based Health-Related Social Media Users. J Med Internet Res 2015; 17(8):e194. DOI:10.2196/jmir.4308
97. Schmeida M., McNeal RS. The telehealth divide: disparities in searching public health information online. J Health Care Poor Underserved 2007; 18(3):637-647. Doi:10.1353/hpu.2007.0068
98. Vanderbilt AA., Wrigth MS. Brewer AE., Murithi LK., Coney, P. Increasing Knowledge and health literacy about preterm Births in Underserved Communities: An Approach to decrease Health Disparities, a pilot study. Glob J Health SCI 2016; 8(1):83-89. Doi.10.5539/gjhs.v8n1p83
99. Viswanath K., Ackerson LK. Race, ethnicity, language, social class, and health communication inequalities: a nationally representative cross-sectional study. PLoS One 2011; 6(1): e14550. Doi:10.1371/journal.pone.0014550
100. Wang JY., Bennett K., Probst J. Subdividing the digital divide: differences in Internet access and use among rural residents with medical limitations. J Med Internet Res 2011; 13(1):e25. Doi:10.2196/jmir.1534
101. West DM., Miller EA. The digital divide in public e-Health: barriers to accessibility and privacy in state health department websites. J Health Care Poor Underserved 2006; 17(3):652-667. Doi:10.1353/hpu.2006.0115
102. Yamin CK., Emani S., Williams DH., Lipsitz SR., Karson AS., Wald JS., Bates DW. The digital divide in adoption and use of a personal health record. Arch Intern Med 2011; 171(6):568-574. Doi:10.1001/archinternmed.2011.34
