# Supplementary material for: Social Health Inequalities and eHealth: A Literature Review With Qualitative Synthesis of Theoretical and Empirical Studies
Source: J Med Internet Res. 2017 Apr 27;19(4):e136. doi: 10.2196/jmir.6731 (PMC5427250; doi:10.2196/jmir.6731)
Supplement: Multimedia Appendix 2 [file jmir_v19i4e136_app2.pdf]

Multimedia Appendix 2. Quality of studies

1. Quality of quantitative studies based on *Quality Assessment Tool for Quantitative Studies*[1]

1. STRONG
2. MODERATE
3. WEAK
N/A. Not Applicable
N/M. Not mentioned

First Author	SELECTION BIAS	STUDY DESIGN	CONFOUNDERS	BLINDING	DATA COLLECTION METHOD	WITHDRAWALS AND DROP-OUTS	INTERVENTION INTEGRITY	ANALYSES	GLOBAL RATING
Anthony [52]	1	2	1	1	1	N/A	N/A	1	1
Bhandari [46]	1	2	1	1	1	N/A	N/A	1	1
Brouwer [78]	1	1	1	1	1	N/A	N/A	1	1
Chilukuri [59]	2	1	1	2	2	N/A	N/A	1	2
Choi [54]	1	1	1	1	2	N/A	N/A	2	1
Chou [79]	1	1	1	1	2	2	N/A	1	1
Chu [62]	2	1	2	1	1	2	2	1	2
Dart [81]	1	1	2	1	2	N/A	N/A	1	2
Dart [80]	2	2	2	1	2	N/A	N/A	1	2
Dudas [82]	1	2	2	1	2	1	N/A	1	2
Eddens [65]	1	1	1	1	1	N/A	N/A	1	1
Feng [40]	1	1	1	1	1	N/A	N/A	1	1
Fowles [84]	2	2	2	2	2	1	N/A	1	2
Geraghty [63]	1	1	2	1	1	N/A	N/A	1	1
Gordon [86]	2	1	1	1	1	2	N/A	1	2
Jones [71]	1	2	2	1	2	2	N/A	1	2
Kontos [67]	2	1	1	1	1	N/A	N/A	1	1
Kontos [12]	2	1	1	1	1	N/A	N/A	1	1
Lee [48]	1	1	2	1	1	2	N/A	1	1
Liszka [89]	2	1	2	1	2	N/A	N/A	1	2

[1] Thomas BH, Ciliska D, Dobbins M, Micucci S. A process for systematically reviewing the literature: providing the research evidence for public health nursing interventions. Worldviews Evid Based Nurs 2004; 1(3):176–84. PMID: 17163895

Lorence [90]	1	1	2	1	1	N/A	N/A	1	1
Lustria [7]	1	1	1	1	1	N/A	N/A	1	1
Massey [49]	1	2	1	1	1	N/A	N/A	1	1
Miller [56]	1	2	1	1	1	N/A	N/A	1	1
Neuenschwander [93]	1	1	1	1	2	N/A	N/A	1	2
Neuenschwander, [34]	2	1	2	1	2	2	2	1	2
Nolke [95]	2	2	1	1	1	N/A	N/A	1	2
Paul [60]	2	2	2	1	2	2	N/A	1	2
Reinwand [6]	2	1	2	1	1	N/A	N/A	1	2
Ryan [57]	2	1	2	2	1	N/M	2	1	2
Sadah [96]	2	2	2	1	2	N/A	N/A	1	2
Sarkar [72]	1	2	2	1	2	N/A	N/A	1	2
Schmeida [97]	N/M	N/M	N/M	N/M	N/M	N/A	N/A	1	3
Vanderbilt [98]	2	1	2	1	1	N/M	N/M	1	2
Viswanath [99]	1	2	1	1	2	N/A	N/A	1	2
Viswanath [10]	1	1	2	1	1	1	1	1	1
Wang [100]	2	1	2	1	1	N/A	N/A	1	2
Yamin [102]	1	1	2	1	1	N/A	N/A	1	1
Zach [47]	2	2	2	2	2	N/A	N/A	1	2
Zhao [48]	1	1	2	1	1	N/A	N/A	1	1

2. Quality of qualitative studies including participatory research action which used a qualitative method based on Letts and all (2007)[2]

1. STRONG
2. MODERATE
3. WEAK
N/A. Not Applicable
N/M. Not mentioned

First Author	Study purpose	Literature	Study design	Qualitative methods	Sampling	Data collection	Data analyses	Overall rigour	Conclusions & implications	Global rating
Apter [64]	2	1	2	2	N/M	2	N/M	3	2	3
Atkinson [61]	1	1	1	1	1	1	1	1	1	1
Atkinson [76]	1	1	1	1	1	1	1	1	1	1
Bacigalupe [45]	1	1	1	1	1	1	1	1	1	1
Bell [77]	1	1	1	1	1	1	1	1	1	1
Gibbons [85]	1	1	2	2	3	3	3	3	2	3
Gordon [53]	1	1	1	N/M	1	N/M	N/M	3	1	3
Kontos [36]	1	1	1	1	1	2	1	1	1	1
Newman [94]	1	1	1	1	1	1	1	2	2	2
West [101]	1	1	1	1	1	2	2	2	2	2

[2] Letts, L., S. Wilkins, M. Law, D. Stewart, J. Bosch, M. Westmorland (2007). Quality Assessment Tool for Qualitative Studies. Critical Review Form – Qualitative Studies, version 2.0, Hamilton (Ontario), University McMaster.

3. Quality of mixed methods studies based on criteria of Schifferdecker & Reed[3] including participatory research action, which used a mixed method.

Y = Yes
N = No
P = partially
N/A. Not Applicable
N/M. Not mentioned

First Author	Identify the study design	Decide on prominence of each data type, analysis, and results	Develop sampling strategies and determine how and when data are collected, analyzed, integrated	Set realistic time requirements	Use software	Review mixed methods articles to generate ideas
Breitenstein [41]	Y	Y	Y	Y	N/M	P
Carlson [50]	Y	P	Y	Y	P	P
Kerr [87]	Y	Y	Y	Y	Y	P
Kukafka [88]	Y	Y	Y	Y	Y	P
Lindsay [33]	P	Y	Y	Y	Y	P
Malone [91]	P	P	P	Y	P	P

[3] Schifferdecker, K. E., Reed, V. A. Using mixed methods research in medical education: Basic guidelines for researchers. Medical Education 2009; 43: 637-644. DOI: 10.1111/j.1365-2923.2009.03386.x

4. Quality of literature reviews based on AMSTAR[4]

Y = Yes
N = No
P = Partially
C = Can not answer
N/A. Not Applicable

First Author	The research question and inclusion criteria should be established before the conduct of the review.	There should be at least two independent data extractors and a consensus procedure for disagreements should be in place.	At least two electronic sources should be searched. The report must include years and databases used. Key words and/or MESH terms must be stated and where feasible the search strategy should be provided. All searches should be supplemented by consulting current contents, reviews, textbooks, specialized	The authors should state that they searched for reports regardless of their publication type. The authors should state whether or not they excluded any reports (from the systematic review), based on their publication status, language etc.	A list of included and excluded studies should be provided.	In an aggregated form such as a table, data from the original studies should be provided on the participants, interventions and outcomes. The ranges of characteristics in all the studies analyzed e.g. age, race, sex, relevant socioeconomic data, disease status, duration, severity, or other diseases should be reported.	'A priori' methods of assessment should be provided (e.g., for effectiveness studies if the author(s) chose to include only randomized, double-blind, placebo controlled studies, or allocation concealment as inclusion criteria); for other types of studies alternative items will be relevant.	The results of the methodological rigor and scientific quality should be considered in the analysis and the conclusions of the review, and explicitly stated in formulating recommendations.	For the pooled results, a test should be done to ensure the studies were combinable, to assess their homogeneity (i.e. Chi-squared test for homogeneity, I^2). If heterogeneity exists a random effects model should be used and/or the clinical appropriateness of combining should be taken into consideration (i.e. is it sensible to combine?).	An assessment of publication bias should include a combination of graphical aids (e.g., funnel plot, other available tests) and/or statistical tests (e.g., Egger regression test).	Potential sources of support should be clearly acknowledged in both the systematic review and the included studies.

[4] Shea BJ, Grimshaw JM, Wells GA, Boers M, Andersson N, Hamel C, et al. Development of AMSTAR: a measurement tool to assess the methodological quality of systematic reviews. BMC Medical Research Methodology 2007; 7:10–10. PMID: 17302989

			registers, or experts in the particular field of study, and by reviewing the references in the studies found.								
Chou [21]	P	P	P	Y	N	Y	Y	C	N	N	N
Dorstyn [18]	Y	Y	Y	Y	N	Y	Y	Y	N	N	N
Gibbons [17]	Y	P	N	N	N	N	N	P	N	N	N
Huxley [20]	Y	C	Y	Y	N	P	N	N	N	N	P
McInnes [22]	Y	Y	Y	Y	N	Y	Y	Y	N	N	Y
Montague [19]	N	N	P	Y	N	N	N	N	N	N	P
Piette [15]	N	N	N	N	N	N	N	N	N	N	Y

*The references that follow are consistents with the references of the article.

6. Reinwand DA, Schulz DN, Crutzen R, Kremers SP, de Vries H. Who Follows eHealth Interventions as Recommended? A Study of Participants' Personal Characteristics From the Experimental Arm of a Randomized Controlled Trial. J Med Internet Res 2015; 17(5): e115. PMID: 25963607
8. Lustria MLA, Smith SA, Hinnant CC. Exploring digital divides: an examination of eHealth technology use in health information seeking, communication and personal health information management in the USA. Health Informatics J 2011 Sep; 17(3): 224–43. PMID: 21937464
11. Viswanath K, McCloud R, Minsky S, Puleo E, Kontos E, Bigman-Galimore C…Emmons, KM. Internet use, browsing, and the urban poor: implications for cancer control. J Natl Cancer Inst Monographs 2013 Dec;2013(47):199–205. PMID: 24395992
12. McAuley A. Digital health interventions: widening access or widening inequalities? Public Health 2014 Dec; 128(12): 1118–20. PMID: 25458115
13. Kontos E, Blake KD, Chou W-YS, Prestin A. Predictors of eHealth usage: insights on the digital divide from the Health Information National Trends Survey 2012. J Med Internet Res 2014;16(7):e172. PMID: 25048379
16. Gibbons MC, Lowry SZ, Patterson ES. Applying Human Factors Principles to Mitigate Usability Issues Related to Embedded Assumptions in Health Information Technology Design. JMIR Hum Factors 2014;1(1):e3. PMID: 27025349
17. Dorstyn DS, Saniotis A, Sobhanian F. A systematic review of telecounselling and its effectiveness in managing depression amongst minority ethnic communities. J Telemed Telecare 2013 Sep;19(6):338–46. PMID: 24163298

18. Montague E, Perchonok J. Health and wellness technology use by historically underserved health consumers: systematic review. Journal of Medical Internet Research 2012 Aug 7;14(4):e78–e78 1p. PMID: 22652979

19. Huxley CJ, Atherton H, Anstey Watkins J, Griffiths F, Watkins JA. Digital communication between clinician and patient and the impact on marginalised groups: a realist review in general practice. British Journal of General Practice 2015;65(641):e813–e821 9p. PMID: 26622034
20. Wen-ying Sylvia Chou, Prestin A, Lyons C, Wen K. Web 2.0 for Health Promotion: Reviewing the Current Evidence. American Journal of Public Health 2013 Jan;103(1):e9–e18 1p. PMID: 23153164
21. McInnes DK, Li AE, Hogan TP. Opportunities for engaging low-income, vulnerable populations in health care: a systematic review of homeless persons' access to and use of information technologies. Am J Public Health 2013 Dec;103 Suppl 2:e11-24. PMID: 24148036
22. Piette JD, Lun KC, Moura LA, Fraser HSF, Mechael PN, Powell J, Khoja, SR. Impacts of e-Health on the outcomes of care in low- and middle-income countries: where do we go from here? Bull World Health Organ 2012 May 1;90(5):365–72. PMID: 22589570
37. Lindsay S, Bellaby P, Smith S, Baker R. Enabling healthy choices: is ICT the highway to health improvement? Health (London) 2008 Jul;12(3):313–31. PMID: 18579630
38. Neuenschwander LM, Abbott A, Mobley AR. Comparison of a web-based vs in-person nutrition education program for low-income adults. J Acad Nutr Diet 2013 Jan;113(1):120–6. PMID: 23092741
39. Morey OT. Digital disparities: the persistent digital divide as related to health information access on the Internet. Journal of Consumer Health on the Internet 2007 Oct;11(4):23–41 19p. http://dx.doi.org/10.1300/J381v11n04_03
40. Kontos EZ, Bennett GG, Viswanath K. Barriers and facilitators to home computer and Internet use among urban novice computer users of low socioeconomic position. J Med Internet Res 2007;9(4):e31. PMID: 17951215
41. Bodie GD, Dutta MJ. Understanding health literacy for strategic health marketing: eHealth literacy, health disparities, and the digital divide. Health Mark Q 2008;25(1–2):175–203. PMID: 18935884
42. Baur C. An analysis of factors underlying e-Health disparities. Camb Q Healthc Ethics 2008;17(4):417–28. PMID: 18724881
44. Feng Y, Xie W. Digital Divide 2.0: The role of social networking sites in seeking health information online from a longitudinal perspective. Journal of Health Communication 2015;20(1):60-68. PMID: 25119019
45. Breitenstein SM, Gross D. Web-based delivery of a preventive parent training intervention: a feasibility study. J Child Adolesc Psychiatr Nurs 2013 May;26(2):149–57. PMID: 23607827
47. Beacom AM, Newman SJ. Communicating health information to disadvantaged populations. Family & Community Health 2010;33(2):152–162 11p. PMID: 20216358
48. Zhao, S. Parental education and children's online health information seeking: beyond the digital divide debate. Soc Sci Med 2009; 69(10): 1501-1505. Doi:10.1016/j.socscimed.2009.08.039

49. Bacigalupe G, Askari SF. E-Health innovations, collaboration, and healthcare disparities: developing criteria for culturally competent evaluation. Fam Syst Health 2013 Sep;31(3):248–63. PMID: 24059273

50. Bhandari N, Shi Y, Jung K. Seeking health information online: does limited healthcare access matter? J Am Med Inform Assoc 2014 Dec;21(6):1113–7. PMID: 24948558
52. Lee C-J, Ramírez AS, Lewis N, Gray SW, Hornik RC. Looking beyond the Internet: examining socioeconomic inequalities in cancer information seeking among cancer patients. Health Commun 2012;27(8):806–17. PMID: 22356137
53. Massey PM. Where Do U.S. Adults Who Do Not Use the Internet Get Health Information? Examining Digital Health Information Disparities From 2008 to 2013. J Health Commun 2016 Jan;21(1):118–24. PMID:26166484
54. Carlson BA, Neal D, Magwood G, Jenkins C, King MG, Hossler CL. A community-based participatory health information needs assessment to help eliminate diabetes information disparities. Health Promotion Practice 2006;7(3):213S–22S 1p. PMID: 16760247
55. Gilmour JA. Reducing disparities in the access and use of Internet health information. A discussion paper. Int J Nurs Stud 2007 Sep;44(7):1270–8. PMID: 16828775
56. Anthony DL, Campos-Castillo C. Do health care users think electronic health records are important for themselves and their providers?: Exploring group differences in a national survey. AMIA Annu Symp Proc 2013:42–9. PMID: 24551321
57. Gordon M, Henderson R, Holmes JH, Wolters MK, Bennett IM. Participatory design of e-Health solutions for women from vulnerable populations with perinatal depression. Journal of the American Medical Informatics Association 2016;23(1):105–109 5p. PMID: 26342219
58. Choi NG, Dinitto DM. The digital divide among low-income homebound older adults: Internet use patterns, eHealth literacy, and attitudes toward computer/Internet use. J Med Internet Res 2013;15(5):e93. PMID: 23639979
60. Miller EA, West DM. Where's the revolution? Digital technology and health care in the Internet age. J Health Polit Policy Law 2009 Apr;34(2):261–84. PMID: 19276318
61. Ryan JG, Schwartz R, Jennings T, Fedders M, Vittoria I. Feasibility of an Internet-based intervention for improving diabetes outcomes among low-income patients with a high risk for poor diabetes outcomes followed in a community clinic. Diabetes Educ 2013 Jun;39(3):365–75. PMID: 23610181
62. Bennett GG. Connecting eHealth with 2-1-1 to reduce health disparities. Am J Prev Med 2012 Dec;43(6 Suppl 5):S509-511. PMID: 23157773
63. Chilukuri N, West M, Henderson JL, Lawson S, Ehsanipoor R, Costigan K…Bennett W. Information and Communication Technology Use Among Low-Income Pregnant and Postpartum Women by Race and Ethnicity: A Cross-Sectional Study. Journal of Medical Internet Research 2015;17(7):1–1 1p. PMID: 26142162
64. Paul CL, Clinton-McHarg T, Lynagh M, Sanson-Fisher RW, Tzelepis F. On-line information and support for supporters and carers of haematological cancer patients: is access an issue? Support Care Cancer 2012 Nov;20(11):2687–95. PMID: 22350593

65. Atkinson NL, Desmond SM, Saperstein SL, Billing AS, Gold RS, Tournas-Hardt A. Assets, challenges, and the potential of technology for nutrition education in rural communities. J Nutr Educ Behav 2010 Dec;42(6):410–6. PMID: 20813589
66. Chu A, Huber J, Mastel-Smith B, Cesario S. "Partnering with seniors for Better Health": computer use and Internet health information retrieval among older adults in a low socioeconomic community. J Med Libr Assoc 2009 Jan;97(1):12–20. PMID: 19159002
67. Geraghty AWA, Torres LD, Leykin Y, Pérez-Stable EJ, Muñoz RF. Understanding attrition from international Internet health interventions: a step towards global eHealth. Health Promot Int 2013 Sep;28(3):442–52. PMID: 22786673
68. Apter AJ. Can patient portals reduce health disparities? A perspective from asthma. Annals of the American Thoracic Society 2014;11(4):608–612 5p. PMID: 24640983

69. Eddens KS, Kreuter MW, Morgan JC, Beatty KE, Jasim SA, Garibay L, Jupka, KA. Disparities by race and ethnicity in cancer survivor stories available on the web. Journal of Medical Internet Research 2009 Oct;11(4):e50–e50 1p. PMID: 19945948
71. Viswanath K, Kreuter MW. Health disparities, communication inequalities, and eHealth. Am J Prev Med 2007 May;32(5 Suppl):S131-133. PMID: 17466818
72. Kontos EZ, Emmons KM, Puleo E, Viswanath K. Communication inequalities and public health implications of adult social networking site use in the United States. J Health Commun 2010;15 Suppl 3:216–35. PMID: 21154095
73. Jones R. Development of a Questionnaire and Cross-Sectional Survey of Patient eHealth Readiness and eHealth Inequalities. Med 2.0 2013; 2(2): e9. DOI: 10.2196/med20.2559
74. Sarkar U., Karter A. J., Liu JY., Adle, NE., Nguyen R., Lopez A., Schillinger D. Social disparities in Internet patient portal use in diabetes : evidence that the digital divide extends beyond access. J Am Med Inform Assoc 2011; 18(3): 318-321. doi: 10.1136/jamia.2010.006015
76. Atkinson NL., Desmond SM., Saperstein SL., Billing AS., Gold RS., Tournas-Hardt A. Assets, challenges, and the potential of technology for nutrition education in rural communities. J Nutr Educ Behav 2009; 42(6): 410-416. Doi:10.1016/j.jneb.2009.09.004
77. Bell AV. "I think about Oprah": social class differences in sources of health information. Qual Health Res 2014; 24(4): 506-516. Doi:10.1177/1049732314524637
78. Brouwer W., Oenema A., Raat H., Crutzen R., de Nooijer J., de Vries NK., Brug J. Characteristics of visitors and revisitors to an Internet-delivered computer-tailored lifestyle intervention implemented for use by the general public. Health Educ Res 2010; 25(4): 585-595. Doi:10.1093/her/cyp063
79. Chou WY., Hunt YM., Beckjord EB., Moser RP., Hesse BW. Social media use in the United States: implications for health communication. J Med Internet Res 2009; 11(4): e48. Doi:10.2196/jmir.1249
80. Dart J. The Internet as a source of health information in three disparate communities. Aust Health Rev 2008; 32(3): 559- 569.
81. Dart, JM, Gallois C. Community desires for an online health information strategy. Aust Health Rev 2010; 34(4): 467-476. Doi:10.1071/ah08719
82. Dudas RA., Crocetti M. Pediatric caregiver attitudes toward email communication: survey in an urban primary care setting. J Med Internet Res 2013; 15(10): e228. Doi:10.2196/jmir.2738
83. Eysenbach G. Poverty, human development, and the role of eHealth. J Med Internet Res 2007; 9(4); e34. Doi:10.2196/jmir.9.4.e34
84. Fowles ER., Gentry B. The feasibility of personal digital assistants (PDAs) to collect dietary intake data in low-income pregnant women. J Nutr Educ Behav 2008; 40(6): 374-377. Doi:10.1016/j.jneb.2007.08.015
85. Gibbons MC., Casale CR. Reducing disparities in health care quality: the role of health IT in underresourced settings. Med Care Res Rev 2010; 67(5 Suppl): 155S-162S. Doi:10.1177/1077558710376202
86. Gordon NP., Hornbrook MC. Differences in Access to and Preferences for Using Patient Portals and Other eHealth Technologies Based on Race, Ethnicity, and Age: A Database and Survey Study of Seniors in a Large Health Plan. J Med Internet Res 2016; 18(3): e50. Doi:10.2196/jmir.5105
87. Kerr C., Murray E., Noble L., Morris R., Bottomley C., Stevenson F . . . Nazareth I. The potential of Web-based interventions for heart disease self-management: a mixed methods investigation. J Med Internet Res 2010; 12(4): e56. Doi:10.2196/jmir.1438
88. Kukafka R., Khan SA., Hutchinson C., McFarlane DJ., Li J., Ancker JS., Cohall A. Digital partnerships for health: steps to develop a community-specific health portal aimed at promoting health and well-being. AMIA Annu Symp Proc 2007:428-432.

89. Liszka HA., Steyer TE., Hueston WJ. Virtual Medical Care: How Are Our Patients Using Online Health Information? Journal of Community Health 2006; 31(5):368-378. Doi:10.1007/s10900-006-9019-3
90. Lorence D., Park H. Study of education disparities and health information seeking behavior. Cyberpsychol Behav 2007; 10(1):149-151. Doi:10.1089/cpb.2006.9977
91. Malone M., While A., Roberts J. Parental health information seeking and re-exploration of the 'digital divide'. Prim Health Care Res Dev 2014; 15(2):202-212. Doi:10.1017/S1463423613000194
92. Munoz RF. Using evidence-based Internet interventions to reduce health disparities worldwide. J Med Internet Res 2010; 12(5):e60. Doi:10.2196/jmir.1463
93. Neuenschwander LM., Abbott A., Mobley AR. Assessment of low-income adults' access to technology: implications for nutrition education. J Nutr Educ Behav 2012; 44(1):60-65. Doi:10.1016/j.jneb.2011.01.004
94. Newman L., Biedrzycki K., Baum F. Digital technology use among disadvantaged Australians: implications for equitable consumer participation in digitally-mediated communication and information exchange with health services. Aust Health Rev 2012; 36(2):125-129. Doi:10.1071/AH11042
95. Nolke L., Mensing M., Kramer A., Hornberg C. Sociodemographic and health-(care-)related characteristics of online health information seekers: a cross-sectional German study. BMC Public Health 2015; 15:31. Doi:10.1186/s12889-015-1423-0
96. Sadah SA., Shahbazi M., Wiley MT., Hristidis VA Study of the Demographics of Web-Based Health-Related Social Media Users. J Med Internet Res 2015; 17(8):e194. DOI:10.2196/jmir.4308
97. Schmeida M., McNeal RS. The telehealth divide: disparities in searching public health information online. J Health Care Poor Underserved 2007; 18(3):637-647. Doi:10.1353/hpu.2007.0068
98. Vanderbilt AA., Wrigth MS. Brewer AE., Murithi LK., Coney, P. Increasing Knowledge and health literacy about preterm Births in Underserved Communities: An Approach to decrease Health Disparities, a pilot study. Glob J Health SCI 2016; 8(1):83-89. Doi.10.5539/gjhs.v8n1p83
99. Viswanath K., Ackerson LK. Race, ethnicity, language, social class, and health communication inequalities: a nationally representative cross-sectional study. PLoS One 2011; 6(1): e14550. Doi:10.1371/journal.pone.0014550
100. Wang JY., Bennett K., Probst J. Subdividing the digital divide: differences in Internet access and use among rural residents with medical limitations. J Med Internet Res 2011; 13(1):e25. Doi:10.2196/jmir.1534
101. West DM., Miller EA. The digital divide in public e-Health: barriers to accessibility and privacy in state health department websites. J Health Care Poor Underserved 2006; 17(3):652-667. Doi:10.1353/hpu.2006.0115
102. Yamin CK., Emani S., Williams DH., Lipsitz SR., Karson AS., Wald JS., Bates DW. The digital divide in adoption and use of a personal health record. Arch Intern Med 2011; 171(6):568-574. Doi:10.1001/archinternmed.2011.34
